# Contrasting bacterial communities in two indigenous *Chionochloa* (Poaceae) grassland soils in New Zealand

**DOI:** 10.1371/journal.pone.0179652

**Published:** 2017-06-28

**Authors:** Jocelyn C. Griffith, William G. Lee, David A. Orlovich, Tina C. Summerfield

**Affiliations:** 1 Department of Botany, University of Otago, Dunedin, New Zealand; 2 Landcare Research, Dunedin, New Zealand; University of Illinois at Urbana-Champaign, UNITED STATES

## Abstract

The cultivation of grasslands can modify both bacterial community structure and impact on nutrient cycling as well as the productivity and diversity of plant communities. In this study, two pristine New Zealand grassland sites dominated by indigenous tall tussocks (*Chionochloa pallens* or *C*. *teretifolia*) were examined to investigate the extent and predictability of variation of the bacterial community. The contribution of free-living bacteria to biological nitrogen fixation is predicted to be ecologically significant in these soils; therefore, the diazotrophic community was also examined. The *C*. *teretifolia* site had N-poor and poorly-drained peaty soils, and the *C*. *pallens* had N-rich and well-drained fertile soils. These soils also differ in the proportion of organic carbon (C), Olsen phosphorus (P) and soil pH. The nutrient-rich soils showed increased relative abundances of some copiotrophic bacterial taxa (including members of the *Proteobacteria*, *Bacteroidetes* and *Firmicutes* phyla). Other copiotrophs, *Actinobacteria* and the oliogotrophic *Acidobacteria* showed increased relative abundance in nutrient-poor soils. Greater diversity based on 16S rRNA gene sequences and the Tax4Fun prediction of enhanced spore formation associated with nutrient-rich soils could indicate increased resilience of the bacterial community. The two sites had distinct diazotrophic communities with higher diversity in *C*. *teretifolia* soils that had less available nitrate and ammonium, potentially indicating increased resilience of the diazotroph community at this site. The *C*. *teretifolia* soils had more 16S rRNA gene and *nifH* copies per g soil than the nutrient rich site. However, the proportion of the bacterial community that was diazotrophic was similar in the two soils. We suggest that edaphic and vegetation factors are contributing to major differences in the composition and diversity of total bacterial and diazotrophic communities at these sites. We predict the differences in the communities at the two sites will result in different responses to environmental change.

## Introduction

Widespread agricultural development of grasslands has made them among the most threatened ecosystems worldwide [1–3]. The cultivation of grasslands can modify both bacterial community structure and nutrient cycling [4–6]. Changes in the bacterial community can arise because different bacterial taxa exhibit different sensitivities to soil conditions including pH [7], C:N ratio [8], moisture [9] and nutrient addition [10,11]. The diversity of soil microbial communities can impact on a range of ecosystem processes; for example, fertilizer addition, elevated temperature and/or CO_2_ have been linked to altered bacterial community composition and community level activity (reviewed in [12]) [13,14].

For a variety of soil types, differences in N availability, drainage and the proportion of organic C have previously been shown to alter both the total bacterial community [15] and the diazotrophic community [16]. For example, C-rich soils have been proposed to favour the growth of copiotrophic bacteria, whereas higher relative abundance of oligotrophs has been observed in soils with lower organic C [17]. In addition, relative abundance of *Acidobacteria* has been shown to be negatively dependent on pH, while abundances of *Actinobacteria* and *Bacteroidetes* were positively dependent on pH [18]. Consistencies in the response of grassland bacterial community composition to nutrient addition have been identified across a broad geographic range; however, local variation in bacterial community structure demonstrates the need for ecosystem and soil-type specific bacterial community analyses [3,17,19].

Approximately 3.6 million hectares (13.4%) of New Zealand’s land is classified as tussock grassland (as of September 2002) [20]. These areas have significant conservation value as they support indigenous biodiversity and provide ecosystem services such as water regulation and erosion control, as well as being culturally significant [20,21]. Indigenous legumes and nitrogen-fixing actinorhizal plants are rare in New Zealand tussock grasslands, and the contribution of free-living nitrogen-fixing bacteria to biological nitrogen fixation is predicted to be ecologically significant [22]. Global estimates of free-living nitrogen fixation in temperate grasslands vary between 0.1–21 kg ha^−1^ year^−1^ with the potential to exceed N inputs from symbiotic nitrogen fixation in environments where symbionts are scarce [23]. Based on the copy number of the *nifH* gene (encoding a subunit of the nitrogenase enzyme that catalyzes nitrogen fixation), the diazotroph community of New Zealand tussock grasslands appears to be sensitive to cultivation [6]. Similarly in grass-scrubland soils in Australia, higher nitrate concentration as a result of frequent livestock grazing was linked to a lower *nifH* gene abundance, supporting the hypothesis that nitrogen-fixation decreases when N is widely available [24]. Similarly, fertilization with both organic and inorganic N decreased nitrogen fixation rates in forest soils [25–27]. However, it is difficult to assess the significance of potential changes in diazotrophs in response to land use without a better knowledge of the diazotrophs in different undisturbed grasslands.

The tall-tussock grasslands of New Zealand are dominated by long-lived perennial snow tussock grasses (bunchgrasses) of the largely endemic genus *Chionochloa* and the short tussock grasslands are generally comprised of *Festuca* and *Poa* [20]. The alpine zone (800–2000 m) in the Murchison Mountains, Fiordland (South Island, NZ) is dominated by *Chionochloa* [28,29]. Diversification within this group resulted in more than 10 species in the region segregated according to altitude, soil conditions and snow lie [30,31]. The edaphic segregation of species influences temperature cues, flowering intensity, costs of seed production and vegetative growth [32,33]. Different species of *Chionochloa* exhibit adaptation to different extremes in natural soil fertility, resulting in distributions that are delimited by soil nutrients [30,32]. Soil N-limitation is a key component differentiating nutrient-rich from nutrient-poor sites in this system [33]. It follows that nitrogen-fixing bacteria must play a significant role in providing biologically available N in the N-limited soils, therefore their diversity and abundance is expected to impact on *Chionochloa* resource allocation and flowering.

This study examined the diversity of the bacterial community in two indigenous grassland soils in the Takahe Valley, Murchison Mountains, at the edaphic extremes occupied by *Chionochloa* snow tussock species on mineral soil [34]. *Chionochloa teretifolia* occupies total N-poor and poorly-drained peaty soils, while *C*. *pallens* occupies N-rich and well-drained fertile soils. These soils also differ in the proportion of organic carbon (C), Olsen phosphorus (P) and soil pH [33]. High-throughput 16S rRNA gene sequencing enables high resolution taxonomic characterization of soil bacterial communities [35–37]. A similar approach, sequencing part of the *nifH* gene and comparing these sequences to the extensive database of *nifH* gene sequences [38,39], enables study of nitrogen-fixing communities. Using this approach we undertook a comprehensive examination of the total bacterial and diazotroph community structure in soils from two undisturbed grassland sites. The aims of this study were to compare the bacterial communities, both total bacterial and diazotroph communities, in the two contrasting indigenous grassland soils and to predict differences in the soil microbial function between the two soil types using Tax4Fun and the 16S rRNA gene dataset.

## Materials and methods

### Site description, sampling and DNA isolation

Soil samples were collected from Takahe Valley, Murchison Mountains, Fiordland National Park, New Zealand (45°14′ S, 167°33′ E) in March 2013. Line transects of approximately 100 m, marked through both the *C*. *pallens* and *C*. *teretifolia* communities, were located approximately 1260 and 1150 m above sea level, respectively. This work was performed under the Department of Conservation permit 38477-FLO. Both species transects were divided into five equal-sized, non-overlapping plots. Within each plot, debris (including coarse roots, stones and loose litter) were removed from the soil surface and 1 cm of the surface soils were collected from five separate locations. From each of the two *Chionochloa* transects, five composite soil samples per species (each composite sample being the result of pooling five individual samples) were collected, and coarse roots and stones were removed. Soil samples were stored in air-tight vials in the dark and then transferred to −20°C. DNA was extracted from 200 mg soil from each composite sample using the MoBio PowerSoil DNA Isolation Kit (MoBio Laboratories) as per manufacturer’s instructions.

### Library construction and sequencing

To determine the bacterial and diazotrophic community composition of the grassland soils, barcoded amplicon libraries were constructed and sequenced. The 16S rRNA (V3–4) region and *nifH* genes were amplified using modified primers 0341F/0785R [40] and 19F/407R [41], respectively. For the first amplification step from soil DNA samples, the primers were synthesized with overhang adapters complementary to the Illumina sequencing primers. A subsequent limited-cycle amplification step was then performed to attach 6-bp multiplexing indices and Illumina sequencing adapters (S1 Table).

PCR amplifications for the bacterial 16S rDNA library were carried out in 50 μl reaction volumes containing final concentrations of: 1× High Fidelity PCR buffer, 2.0 mM MgSO_4_, 200 μM dNTPs, 200 nM each of 0341F and 0785R primers, 0.05% of bovine serum albumin (BSA) and 1 U Platinum Taq DNA Polymerase High Fidelity (Thermo Fisher Scientific). The reactions were held at 94°C for 2 min, followed by 35 cycles of 94°C for 30 s, 55°C for 30 s, 68°C for 40 s and a final extension of 68°C for 2 min. Amplicons were purified using Agencourt AMPure XP beads (Beckman Coulter) and eluted in 30 μl of UltraPure DNAse/RNAse-free distilled water (Thermo Fisher Scientific) and quantified using the Qubit dsDNA High Sensitivity Assay (Thermo Fisher Scientific). Second round PCR was performed with 2 ng of purified amplicons as template using the same reaction concentrations as the first round. The PCR conditions were 95°C for 20 s, followed by 10 cycles of 95°C for 20 s, 55°C for 20 s, 68°C for 20 s and a final extension of 68°C for 40 s. Amplicons were again purified using Agencourt AMPure XP beads and eluted in 30 μl of nuclease-free distilled water.

PCR amplifications for the *nifH* library were performed using the universal primer set 19F/407R [41], which has been predicted to have a high coverage of *nifH* sequences based on *in silico* testing and also amplified from soil DNA [42]. The 50 μl reaction mixtures contained final concentrations of 1× High Fidelity PCR buffer, 2.0 mM MgSO_4_, 200 μM of each dNTP, 200 nM each of 19F and 407R primers, 0.05% of BSA and 1 U Platinum Taq DNA Polymerase High Fidelity. The reactions were held at 94°C for 2 min, followed by 30 cycles of 94°C for 30 s, 51°C for 30 s, 68°C for 40 s and a final extension of 68°C for 2 min. Amplicon purification, second round of PCR amplification, and the subsequent purification and elution steps were the same as the 16S rDNA library.

Amplicons were run on the Agilent 2100 Bioanalyzer (Agilent Technologies) for size assessment. A paired-end 250-bp sequencing run was performed on the Illumina MiSeq (New Zealand Genomics Limited). Sequences are deposited in the NCBI Sequence Read Archive (SRP053025) under accession numbers SRX2012063–SRX2012072 (16S rRNA) and SRX2014251–SRX2014260 (*nifH*).

### Bioinformatic analyses

Samples were demultiplexed using bcl2fastq Conversion Software (Illumina). Sequence data were processed and quality controls were performed through a combination of the UPARSE, mothur and QIIME pipelines [43–45]. Paired-end reads were joined to build the 16S rRNA and *nifH* gene sequences using the -fastq_mergepairs option of USEARCH v7.0.1090 [45]. Quality control processing was carried out via the UPARSE pipeline using -fastq_filter command, and sequences with more than 0.5 expected errors were removed (-fastq_maxee 0.5) [45]. Putative frameshifts in *nifH* sequences were detected and corrected with the FrameBot tool (http://fungene.cme.msu.edu/FunGenePipeline/) [46]. Further processing was performed on mothur v.1.34.0 [45] using the command screen.seqs to include only sequences that were between 400–500 bp and 300–400 bp for the 16S rRNA and *nifH* gene sequences, respectively. Reads were clustered into OTUs using the UPARSE pipeline based on 97% sequence identity. Chimeric sequences were identified and removed using the uchime_ref command [45] against the ChimeraSlayer “Gold” database from the Broad Microbiome Utilities version microbiomeutil-r20110519 [47] for 16S rRNA sequences and using the reference-free de novo chimera detection mode for *nifH* sequences [45]. Taxonomic assignment for the 16S rRNA sequence data were made in QIIME against the SILVA 119 database using the Ribosomal Database Project (RDP) Classifier [48]. An aligned *nifH* gene database [49] using the default assignment method in QIIME (UCLUST) was used for *nifH* gene taxonomic assignment. Sequences belonging to *nifH* clusters IV and V that code for genes involved in bacteriochlorophyll and chlorophyll biosynthesis [50] and thus are not related to nitrogen fixation were removed. Singleton OTUs, archaea and chloroplast sequences were removed from the 16S rRNA gene dataset. For the ten soil samples from *C*. *teretifolia* and *C*. *pallens*-dominated grasslands, a total of 4 893 446 and 2 024 357 reads were obtained through high-throughput sequencing of the 16S rRNA and *nifH* genes, respectively. Following the removal of singletons, low-quality sequences and *nifH* sequences belonging to clusters IV and V, 434 260 sequences remained for downstream analyses (S2 Table). This resulted in a total of 19 219 16S rDNA- and 547 *nifH*-based OTUs. Rarefaction analyses at 97% similarity clustering showed that both 16S rRNA and *nifH* gene libraries reached their plateau (S1 Fig). Subsamples of 198 769 and 9658 random 16S rRNA and *nifH* gene sequences, respectively, were analyzed from each sample to compare diversity and richness across sample sites. Tax4Fun [51] was used to predict functional profiles from 16S rRNA genes within the R environment [52].

To construct the *nifH* phylogenetic tree, *nifH* sequences were first clustered at 97% similarity OTUs containing > 100 sequences and their closest sequence matches were retrieved from GenBank using the NCBI BLASTX search. The *nifH* sequences were aligned with MUSCLE v3.8.31 under default parameters [45] and visually inspected in Geneious R9 [53]. A phylogenetic tree of the nucleotide sequences was constructed with Bayesian inference using MrBayes v3.2.5 [54,55] with four separate chains for 72.5 × 10^6^ generations. Sampling frequency was set to every 100 generations and the first 25% of samples were discarded (“burnin”). The Bayesian analysis was run using a GTR + I + Γ (lset nst = 6, rates = invgamma) model of substitution as selected by MrModelTest 2.3 [56] and was run until the standard deviation of split frequencies was < 0.01. The tree was displayed on FigTree and heatmaps were generated with the *heatmap*.*2* function of the ‘gplots’ package for R [52].

Phylogenetic trees of the class *Ktedonobacteria* (*Chloroflexi*) and the *Nitrosomonadaceae* family (*Betaproteobacteria*) were constructed using a similar method to the *nifH* tree with the exceptions described below. Closest sequences matches were retrieved form GenBank using the BLASTN search. The nucleotide alignments were visually inspected and gaps were excluded for phylogenetic analysis, and the Markov chains were run for a total of 96 and 2 million generations for *Ktedonobacteria* and *Nitrosomonadaceae* Bayesian analyses, respectively.

### Real-time PCR assay

Quantitative real-time PCR (qPCR) was performed using a MiniOpticon Real-Time PCR System (Bio-Rad). To estimate total bacterial abundance in soils we targeted the 16S rRNA gene using modified Earth Microbiome Project recommended primers 515f and 806rB [57]. qPCR targeting the *nifH* gene was performed using the nifH-F (5′-AAAGGYGGWATCGGYAARTCCACCAC-3′) and nifH-R (5′- TTGTTSGCSGCRTACATSGCCATCAT-3′) as these primers have been predicted to have a lower affinity for non-*nifH* sequences from cluster IV [42,58]. Reaction mixtures of 10 μl were composed of 1 μl gDNA template (1 in 10 dilution, ranging from 1–8 ng), 5 μl of 2 × SensiFAST SYBR No-ROX mix (Bioline) and 400 nM of each primer. The cycling conditions for both genes was as follows: 95°C for 3 min and 40 cycles of 95°C for 5 s, 60°C for 10 s, 72°C for 15 s, and data acquisition at 80°C for 15 s. All qPCR assays were carried out in triplicate and included negative (no template) controls. Melt-profile analyses were performed to confirm the absence of primer dimer or non-specific amplification products. Standard curves were performed for each primer pair using 10-fold serial dilutions of linearized plasmids containing target 16S rRNA or *nifH* gene fragments as the insert. Linearized plasmids were quantified using Qubit dsDNA High Sensitivity Assay Kit and a Qubit 2.0 fluorometer. PCR efficiencies were calculated from standard curves, these were 86% and 91% for 16S rRNA and *nifH* genes, respectively.

### Statistical analysis

One-way analysis of variance (ANOVA) was used to determine whether there was a significant effect of sample site on diversity or richness of 16S rRNA and *nifH* gene sequences. Alpha and beta diversity were calculated, and pairwise distances using weighted and unweighted UniFrac matrices [59] were used to generate Principal Coordinates Analysis (PCoA) plots [44]. Permutational multivariate analysis of variance (PERMANOVA) [60] tests were performed to test if beta diversity differed significantly among communities in different sites (permutations = 999). The heatmap was generated using the *heatmap*.*2* function of the ‘gplots’ package [61] for R [52]. Only the top 34 most abundant 16S rRNA gene-based families were used to generate the heatmap. Clustering was accomplished using average linkage method in the *hclust* function.

## Results and discussion

### Alpha diversity and richness based on 16S rRNA and *nifH* gene sequences differ between the two sites

**Fig 1:**
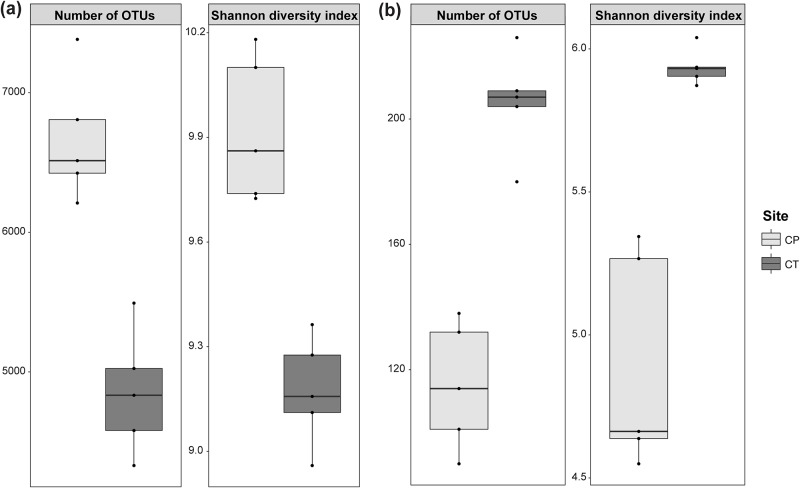
Boxplots of sample richness and diversity at a genetic distance of 3% in *Chionochloa pallens* and *Chionochloa teretifolia* grassland soils based on (a) 16S rRNA and (b) *nifH* gene sequences. Number of unique OTUs and Shannon diversity indices were calculated at 97% similarity for 198 769 and 9658 subsampled 16S rRNA and *nifH* gene sequences, respectively.

Both the 16S rRNA gene and *nifH* gene sequences showed more variable α-diversity between the two sites than between samples collected from within either site. For the 16S rRNA gene sequences the number of OTUs (6667 ± 453 [mean ± SD] versus 4852 ± 444; ANOVA, *P* < 0.001) and the Shannon diversity index (9.9 ± 0.2 versus 9.1 ± 0.3; ANOVA, *P* < 0.001) were higher in *C*. *pallens* soils compared to *C*. *teretifolia* soils (Fig 1a). Conversely, in the diazotrophic community the number of OTUs (115 ± 20 versus 205 ± 17; ANOVA, *P* < 0.001) and Shannon diversity index (4.9 ± 0.4 versus 5.9 ± 0.1; ANOVA, *P* < 0.001) were significantly lower in the *C*. *pallens* soils compared to *C*. *teretifolia* soils (Fig 1b). As expected the diversity and richness of the diazotroph community was lower than that of the total bacterial community. However, it was unexpected that the higher diversity and richness of the diazotrophic community was at the *C*. *teretifolia* site where there was lower diversity and richness of the bacterial community based on 16S rRNA gene sequences.

### Bacterial communities differ in *C*. *pallens* and *C*. *teretifolia* grassland soils

**Fig 2:**
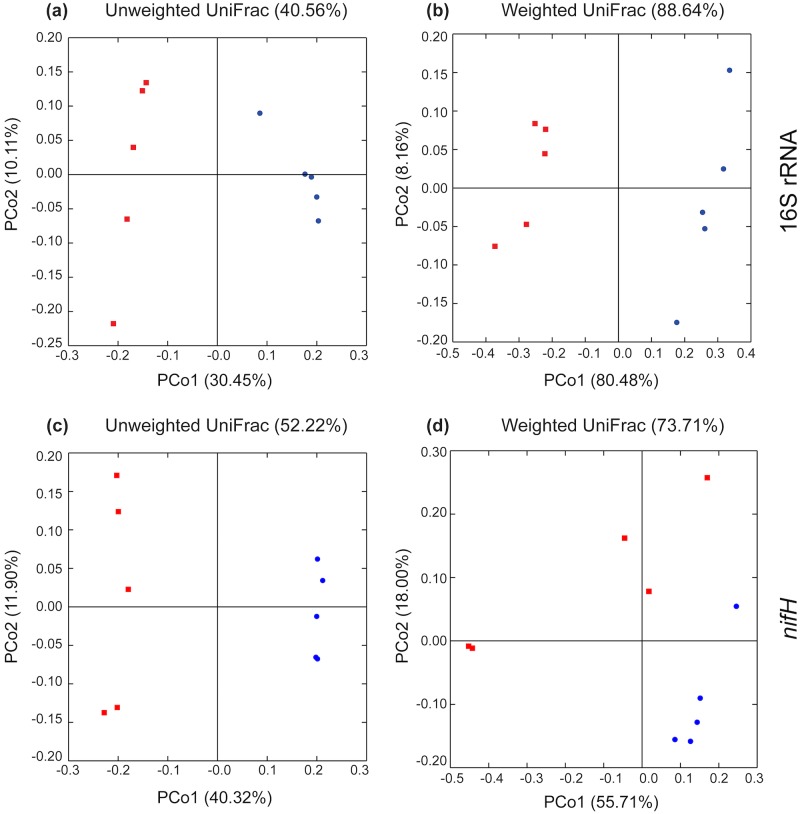
Principal coordinates analysis plots illustrating beta diversity of total bacterial communities based on 16S rRNA gene sequences (a and b) and nitrogen-fixing community based on *nifH* gene sequences (c and d) at *Chionochloa pallens* (CP) and *Chionochloa teretifolia* (CT) sample sites. UniFrac distance matrices are generated from OTUs of 97% identity. Unweighted plots (a and c) are based on the presence or absence of OTUs; weighted plots (b and d) are based on the relative abundance of OTUs. Red dots are samples from *C*. *pallens* sites and blue dots are from *C*. *teretifolia* sites.

Principal coordinates analyses (PCoA) based on the unweighted and weighted UniFrac distances of the 16S rRNA gene sequences showed that the *C*. *pallens* samples were distinct from those of the *C*. *teretifolia* samples (Fig 2a and 2b). PERMANOVA showed that there was a significant difference in the bacterial communities at the two sites (Pseudo-*F* = 21.2323, *P* = 0.009 for weighted; Pseudo-*F* = 3.4357, *P* = 0.011 for unweighted analyses). Comparison of *nifH* sequences showed *C*. *pallens* soil samples were separated from the *C*. *teretifolia* soil samples in the unweighted UniFrac analysis, but the diazotrophic communities from the two sites were not so clearly separated using weighted UniFrac analysis (Fig 2c and 2d). This was confirmed using PERMANOVA (Pseudo-*F* = 3.9344, *P* = 0.01 for weighted; Pseudo-*F* = 5.912, *P* = 0.009 for unweighted analyses). For both 16S rRNA and *nifH* sequences, Bray-Curtis dissimilarities for intrasite samples were significantly lower than intersite samples (*P* < 0.0001 for 16S rRNA and *nifH* sequences, two-tailed, two-sample *t* test) (S3 Table).

### Differences in abundance and diversity of bacterial phyla and *nifH* clusters in the two *Chionochloa* grassland soils

**Fig 3:**
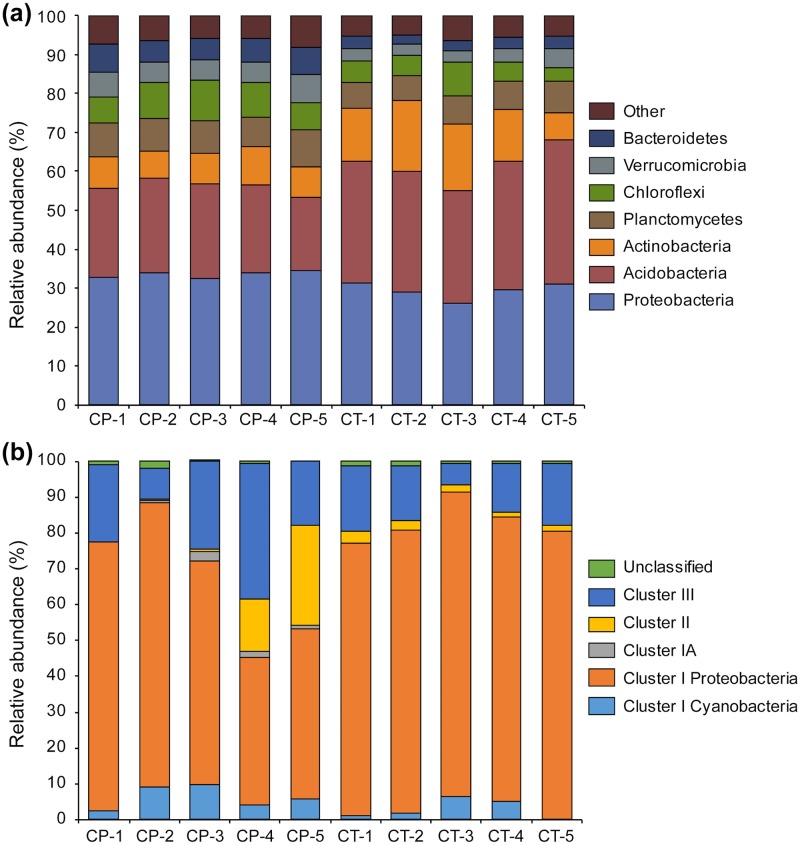
Taxonomic assignments of sequences from *Chionochloa pallens* (CP) and *Chionochloa teretifolia* (CT) grassland soils based on (a) 16S rRNA and (b) *nifH* gene sequences. Only dominant bacterial phyla (over 1%) of 16S rRNA gene sequences are shown. The *nifH* clusters are shown as previously designated [62].

The dominant phyla in all samples were *Acidobacteria*, *Actinobacteria*, *Bacteroidetes*, *Chloroflexi*, *Planctomycetes*, *Proteobacteria* and *Verrucomicrobia* (Fig 3). These were previously reported to be the most abundant phyla in a study of 25 grassland soils worldwide [3]. Less abundant phyla (< 1% of all classified sequences; 20 phyla) are shown in S4 Table. In *C*. *teretifolia* sites compared to *C*. *pallens* sites there was higher ranking of *Acidobacteria* and *Actinobacteria* and lower ranking of *Proteobacteria*, *Planctomycetes*, *Chloroflexi*, *Bacteroidetes* and *Verrucomicrobia* (S4 Table). Several of the differences between the *C*. *pallens* and *C*. *teretifolia* sites were consistent with other findings that have reported an increase of copiotrophic (or *K*-selected) or faster-growing bacterial taxa under increased available ammonium and nitrate [3,15,17]. This included increased abundance of putative copiotrophic taxa, i.e., *Alphaproteobacteria*, *Betaproteobacteria* and *Bacteroidetes* in the *C*. *pallens* sites compared to *C*. *teretifolia* sites (S4 Table).

In addition, the increased relative abundance of *Acidobacteria* in the *C*. *teretifolia* sites was consistent with identification of this phylum as oligotrophic (or *r*-selected). *Acidobacteria* have increased abundance in N-poor soils; this has been suggested to be due to their ability to metabolize less fertile and recalcitrant organic C that is largely unavailable to other bacteria [17,19,63]. This distribution of phyla supports the model of a shift from oligotroph to copiotroph communities based on low to high resource availability [15,17]. However, *Actinobacteria* which have been identified as copiotrophic [3,17], exhibited higher relative abundance in *C*. *teretifolia* soils (13.8 ± 3.9%) compared to *C*. *pallens* soils (8.1 ± 1.0%) (S4 Table). Many other soil characteristics differ between the two sites, which may contribute to the differences in bacterial community. For example, greater relative abundance of *Actinobacteria* was found in *C*. *teretifolia* soils, which have a higher C:N ratio compared to *C*. *pallens* soils [33]. Actinobacterial abundance in soil was previously positively correlated with total microbial biomass and C:N ratio in grassland soils [35,64].

Increased diversity of 16S rRNA genes per genome within certain bacterial taxa that contain high copy numbers of 16S rRNA can overestimate diversity estimates using OTU clustering [65]. In particular, member of the phyla *Firmicutes*, *Gammaproteobacteria* and *Bacteroidetes* have been reported to have diverse multiple copies of 16S rRNA gene per genome [65] and their higher relative abundance in *C*. *pallens* soils might attribute to the higher diversity in these soils.

### Differences in bacterial families with high relative abundance in the *C*. *pallens* and *C*. *teretifolia* soils

**Fig 4:**
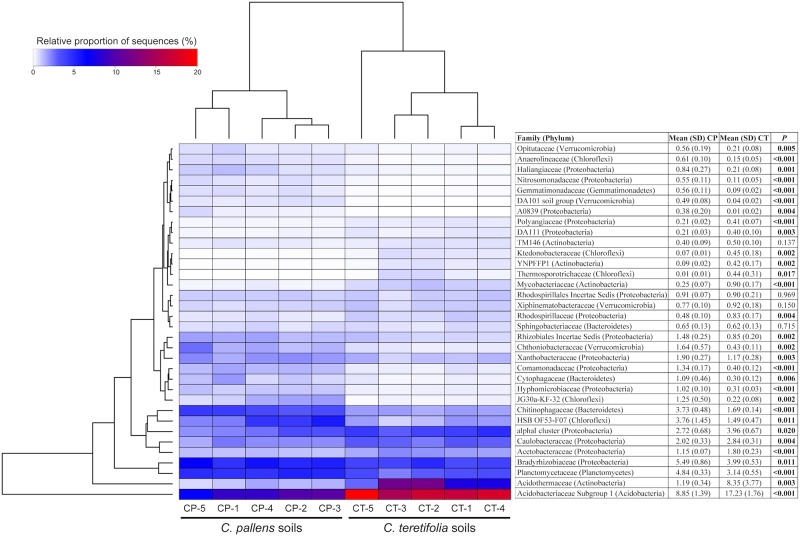
Heatmap of most abundant families in each sample based on 16S rRNA gene sequences. The heatmap indicates relative proportions of most abundant families within each sample. Double dendrograms are based on average linkage hierarchical clustering.

Among the most abundant families, *Acidobacteriaceae* Subgroup 1 (*Acidobacteria*) and three families belonging to *Actinobacteria*, i.e., *Acidothermaceae*, *Mycobacteriaceae* and YNPFFP1, were significantly more abundant in *C*. *teretifolia* grassland soils (*P* < 0.01 for all families; Fig 4). The mean abundance of *Acidobacteriaceae* Subgroup 1 in the low pH *C*. *teretifolia* soils (mean of pH 4.5 [33]) was more than double that in *C*. *pallens* soils (mean of pH 5.2; Fig 4). Previously the abundance of many acidobacterial subgroups, including subgroup 1, were known to correlate negatively with soil pH [66]. Within the *Acidothermaceae* family (*Actinobacteria*) and *Actinobacteridae* subclass, the genus *Acidothermus* (the most abundant genus across all soil samples) was more abundant in *C*. *teretifolia* soils (8.4 ± 3.4%) compared to *C*. *pallens* soils (1.2 ± 0.3%) (ANOVA, *P* = 0.003). However, the opposite pattern was observed in 88 soils across North and South America, where the relative abundance of *Actinobacteridae* had decreased with increasing pH (between pH < 4 and >8) [18], indicating that other soil variables in addition to pH may be contributing to the changes in the relative abundances of *Actinobacteria*. The most abundant Proteobacterial families were *Bradyrhizobiaceae*, *Acetobacteraceae* and *Caulobacteraceae*, the latter two were relatively more abundant in *C*. *teretifolia* soils while *Bradyrhizobiaceae*, which includes four genera containing nitrogen-fixing bacteria [67], was relatively enriched in *C*. *pallens* soil (Fig 4).

### OTUs unique to either the *C*. *pallens* or *C*. *teretifolia* soils

Comparative analysis of the bacterial community in both grassland soils revealed 1118 and 334 of the 16S rRNA gene OTUs were present in only *C*. *pallens* or *C*. *teretifolia* soils, respectively. Many (over 40%) of the sequences that were absent from *C*. *pallens* soils were classified as *Chloroflexi* in the class *Ktedonobacteria*. Phylogenetic analyses of the most abundant (> 100 reads) *Ktedonobacteria* OTUs identified four clades consisting of 20 OTUs that were only present in *C*. *teretifolia* soils, and none of these were closely related to any previously identified *Ktedonobacteria* strains (S2 Fig; marked with bars). The closest cultured relative of many of these OTUs was *Ktedonobacter racemifier*, which has been described as an aerobic heterotroph that grows well at moderately acidic (pH 4.8–6.8) and microaerophilic conditions [68,69]. The *C*. *teretifolia* soils are often poorly drained [29,33] and thus microaerobic conditions are likely to develop. The unique *Ktedonobacter* OTUs in the *C*. *teretifolia* soils might indicate an increase in closely related new or rare species that might be capable of growth in microaerobic conditions. This is also consistent with a previous report of positive correlation between increased relative abundance and diversity of *Chloroflexi* that was proposed to indicate recruitment of new species or increase in rare species [70].

In contrast, sequences that were only present in *C*. *pallens* soils consisted of approximately 20% *Proteobacteria* (mostly *Betaproteobacteria*, family *Nitrosomonadaceae*), 17% *Chloroflexi* (unclassified beyond the phylum level) and 9% each of *Planctomycetes* and *Verrucomicrobia* (*Spartobacteria*). The absence of the family *Nitrosomonadaceae* in more acidic *C*. *teretifolia* soils might reflect selection against ammonium-oxidizing bacteria as they prefer a higher pH range (pH 6–9) for growth and nitrification [71,72]. However, phylogenetic analyses of the abundant OTUs belonging to the family *Nitrosomonadaceae* did not group the OTUs with closely related to known ammonium-oxidizers in the genera *Nitrosospira* and *Nitrosomonas* (S3 Fig).

The relative abundance of *Planctomycetes* was significantly higher in *C*. *pallens* grassland soils (S4 Table), which has higher nitrate N levels compared to *C*. *teretifolia* soils [33]. Increased abundance and diversity of *Planctomycetes* has previously been correlated with higher nitrate concentrations as a result of fertilization and cultivation [73,74]. Despite being the most common verrucomicrobial class in soil [75], only one representative from *Spartobacteria* has been cultured to date [76]. Therefore, the ecological relevance of the increased relative abundance of *Spartobacteria* in *C*. *pallens* grassland soils is not known.

### Differences in putative diazotrophic communities between the two grassland soils

**Fig 5:**
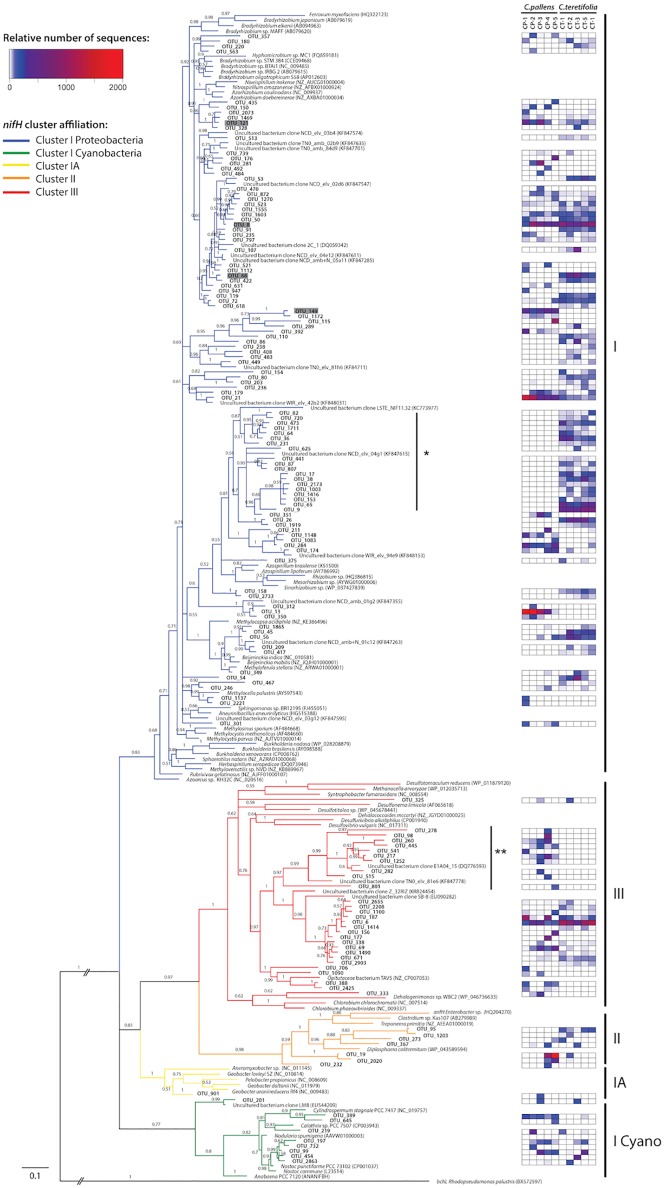
Bayesian phylogenetic tree inferred from 97% nucleotide similarity clustered *nifH* gene sequences and their nearest relatives grouped into canonical *nifH* clusters. Heatmap illustrating the relative number of sequences for each OTU is shown, with the lower values of the dataset coloured in blue and the highest value in bright red. Numbers associated with nodes are Bayesian posterior probabilities. GenBank accession numbers are indicated next to reference taxa. For clarity purposes only OTUs with 100 sequences and more have been included in this tree.

Sequences were distributed into *nifH* clusters I, II and III (Fig 3b) as defined previously: cluster I includes *nifH* sequences that encode the conventional FeMo nitrogenases found in *Cyanobacteria*, *Alpha*-, *Beta*- and *Gammaproteobacteria*; cluster II contains sequences belonging to the alternative FeV and FeFe nitrogenases and some archaea; cluster III includes *nifH* sequences from anaerobic bacteria such as *Clostridia* and *Deltaproteobacteria* [62]. In both *C*. *pallens* and *C*. *teretifolia* soils the majority of *nifH* gene sequences belonged to cluster I *Proteobacteria* (61.1 ± 16.6 and 79.9 ± 3.2%, respectively); however, the majority of OTUs did not form clades with sequences from nitrogen-fixing soil genera such as *Azospirillum*, *Bradyrhizobium*, *Burkholderia*, *Methylocystis* and *Methylocella* (Fig 5). The largest difference between the sites was an increase in the number of *nifH* cluster I Proteobacterial OTUs in *C*. *teretifolia* soils (ANOVA, *P* = 0.038; Fig 3b). In contrast, one of the most abundant OTUs (OTU_13), represented 5.8% of *nifH* sequences and was almost entirely only found in *C*. *pallens* soils (Fig 5). This OTU was in a strongly supported group with an uncultured nitrogen-fixing bacterium from a pine forest site (KF847355; [77]). These were contained in a group with sequences representing members of the genera *Azospirillum*, *Mesorhizobium* and *Sinorhizobium* (Fig 5). Overall, the high abundances of *nifH* sequences related to *Azospirillum* and *Bradyrhizobium* (Fig 5) across both grassland soils are in accordance to the dominance of diazotrophs within with these genera in soils [78]. In addition, the most abundant 16S rRNA gene-based OTU appear to belong to *Bradyrhizobium* (*Alphaproteobacteria*). Several of the most abundant *nifH* OTUS are related to *Bradyrhizobia* (shaded in gray; Fig 5) that share 91–99% sequence identity (BLASTN) may be represented by the same 16S rRNA-based OTU. The *nifH* genes are frequently more variable than 16S rRNA genes, for example strains with < 3% sequence dissimilarity for the 16S rRNA genes have been reported to have up to 23% dissimilarity in their *nifH* genes [39].

The *nifH* cluster I *Cyanobacteria* showed trends of increased abundance in *C*. *pallens* (6.2 ± 3.2%) versus *C*. *teretifolia* (2.9 ± 2.8%) sites (Fig 3). In both sites, *nifH* sequences comprised of the filamentous heterocyst-forming genera *Nostoc*, *Cylindrospermum* and *Calothrix*. One OTU (OTU_389), which was in a well-supported clade with the heterocystous nitrogen-fixer *Cylindrospermum stagnale* PCC 7417 and OTU_645, was found in all *C*. *pallens* sites but was present at low abundance in only two *C*. *teretifolia* sites (Fig 5). Higher phosphate concentrations in *C*. *pallens* soils [33] could influence nitrogen-fixation activity, and it has been shown that phosphate inoculations increased the activity of nitrogen-fixing cyanobacteria in a salt marsh [79]. Similar cyanobacteria from the genera *Nostoc* and *Nodularia* have already been described as important sources of N input in boreal N-limited forest [80] and alpine ecosystems [81]. In fact, non-symbiotic heterocystous bacteria, primarily *Nostoc* sp., were attributed as the main contributors of biologically-fixed N in a natural temperate grasslands lacking legumes [82]. However, these represented only a small proportion of *nifH* OTUs in this study.

The *nifH* cluster III is composed of anaerobic organisms [38], and this cluster has been estimated to constitute around 11% of the *nifH* pool in soils [49]. There was increased relative abundance of cluster III *nifH* sequences in *C*. *pallens* sites (22.1 ± 10.7%) compared to *C*. *teretifolia* sites (14.1 ± 5.0%) (Fig 3), and a large proportion of *nifH* cluster III sequences was found predominantly in *C*. *pallens* samples (Fig 5). This diverse group consisted of at least nine OTUs that were not closely related to any known cultured diazotroph but is closely related to an uncultured soil bacterium isolated from white spruce rhizospheres (DQ776593; Fig 5) [83]. The most abundant single OTU (OTU_6; Fig 5), is in a well-supported group with other OTUs and uncultured bacterium (EU090282) from an acidic peat bog. Sister to this group is a well-supported clade containing *Opitutacaea* bacterium TAV5 (Fig 5). There were ten *nifH* OTUs (> 100 sequences) that were found exclusively in *C*. *pallens* soils (Fig 5).

Cluster II represented a smaller percentage of sequences in this study (8.8 ± 12.3% in *C*. *pallens* and 2.2 ± 5.5% in *C*. *teretifolia* sites). This cluster contains genes encoding the alternative nitrogenases with FeV and FeFe metal clusters (encoded by *vnfH* and *anfH* genes, respectively) and *nifH* sequences of some methanogenic archaea [38]. An earlier survey in NZ soils proposed that nitrogen-fixing *Clostridia* are more abundant in soils with a higher N availability [84]. Consistent with this, one of the most abundant *nifH*-based OTUs (OTU_19) in cluster II was more abundant in *C*. *pallens* soils (Fig 5). However, as this OTU was only distantly related to the genera *Diplosphaera*, *Treponema* and *Clostridium* (Fig 5), it is difficult to ascertain the phylogenetic affiliation of this OTU and other sequences in cluster II.

The *nifH* sequences belonging to cluster IA, which contains predominantly obligate anaerobes, were found exclusively in *C*. *pallens* soils (Fig 3). The single OTU (OTU_901) in cluster IA with > 100 sequences was related to *Geobacter* sp. and *Pelobacter* sp. (*Deltaproteobacteria*) (Fig 5). The *Geobacter*-*Pelobacter* group is anaerobic and the ability to fix N_2_ in nutrient-deficient environments has seen various *Geobacter* spp. thrive in a wide variety of ecosystems, from temperate forests [77] to extreme environments such as Antarctic zones and alpine glaciers [85,86]. Therefore, it is surprising that these bacteria might be more common in the generally higher nutrient and well-drained *C*. *pallens* soils.

### Abundance of 16S rRNA and *nifH* genes in *C*. *pallens* and *C*. *teretifolia* soils

A greater number of OTUs and higher Shannon diversity index indicated that *nifH* gene diversity was greater in *C*. *teretifolia* soils. However, the result was not significant when we used *nifH* OTUs that were clustered based on 90% identity, indicating the diversity in *C*. *teretifolia* soils resulted from closely related bacteria. To determine whether the abundance of the nitrogen-fixing community differed in these grassland soils, we quantified the abundance of bacterial and archaeal 16S rRNA and *nifH* genes. Using qPCR the number of copies of the 16S rRNA and *nifH* genes were calculated per g of soil. Gene copy numbers of bacterial 16S rRNA genes were significantly higher in *C*. *teretifolia* soils compared to *C*. *pallens* soils (ranging from 4.9 × 10^6^–3.8 × 10^7^ copies g^-1^, and 3.3 × 10^7^–1.2 × 10^8^ copies g^-1^ soil for *C*. *pallens* and *C*. *teretifolia* soils, respectively) (ANOVA, *P* <0.01). Gene copy numbers of *nifH* ranged from 8.0 × 10^4^–4.1 × 10^5^ copies g^-1^ in *C*. *pallens* soils and 7.2 × 10^5^–2.0 × 10^6^ copies g^-1^ soil in *C*. *teretifolia* soils (ANOVA, *P* <0.001). However, the ratio of *nifH* to 1000 copies of 16S rRNA gene copies was similar in the two soil types, i.e., 15.2 ± 4.3 and 16.6 ± 4.3 in *C*. *pallens* and *C*. *teretifolia* soils, respectively (S4 Fig). This indicated an increased number of bacterial and archaeal cells per g soil in *C*. *teretifolia* as well as an increase in diazotrophs per g soil, but no change in the proportion of diazotrophs in the community between the two soil types.

Microbial biomass carbon and total N were two of the drivers of *nifH* abundance in managed and remnant (minimally disturbed) soils in Australia [87]. Therefore, it is suggested that soil N availability not only impacts the above-ground vegetation productivity [33], but also the diazotroph community. However, we did not find support for the hypothesis that greater abundance of diazotrophs based on *nifH* to 16S rRNA gene ratio would be found in soils with higher N availability (*C*. *pallens* soils). Agricultural soils with higher nitrate and ammonium levels have been reported to contained lower *nifH* gene copy numbers g^-1^ dry soil following fertilization [78]. Ammonium is known to inhibit nitrogenase [88] and thereby could be causing a decrease of diazotrophs in *C*. *pallens* soils. Higher N availability might reflect differences in the *nifH* transcript levels or different N mineralization rate of the bacterial communities.

### Predicted functional analysis using Tax4Fun

To assess how variation in edaphic properties may influence the functional profiles of soil bacterial communities in these grassland soils, we used the approach of inferred metagenomics using Tax4Fun from 16S rRNA data [51]. Tax4Fun analysis revealed many KEGG Ortholog (KO) profiles related to sporulation among the 50 most differentially abundant KOs, these were significantly more abundant in *C*. *pallens* soils (*P* < 0.01, all analyses, Mann-Whitney U-test; S5 Table). Higher relative abundances of spore-forming genera, i.e., *Clostridium*, *Bacillus*, *Paenibacillus* and *Anoxybacillus*, were found in *C*. *pallens* soils (data not shown). Dormancy in both spore-forming and non-spore-forming bacteria is thought to provide the opportunity for functionally redundant species to co-exist [89,90]; and therefore, may buffer the impacts of changing environmental conditions and disturbances [91]. This could lead to the maintenance of ecosystem and community functioning in spite of shifts in soil microbial community composition, which can have major implications for fragile ecosystems.

## Conclusions

In this study, we found that both the total bacterial community and diazotrophic community were distinct between two pristine grassland soils that are dominated by different but closely related *Chionochloa* species in New Zealand. Total bacterial diversity was greater in the *C*. *pallens* soils, which had higher nutrient availability [33], and this is consistent with previous observations in grassland soils [92]. Bacterial phyla exhibited ecological traits according to the availability of nutrients in soils as previously predicted [15] to an extent. We found that the more acidic, nutrient poor *C*. *teretifolia* soils harboured a greater abundance of oligotrophs, i.e., *Acidobacteria*, compared to copiotrophs. On the other hand, greater relative abundance of *Proteobacteria*, *Bacteroidetes* and *Firmicutes* were found in *C*. *pallens* soils; consistent with slightly higher pH and higher N availability soils favouring copiotrophs over oligotrophs. It should be noted many additional factors differ between the two soil types that may contribute to the differences in the bacterial communities. The increased diversity in the *C*. *pallens* soils might be expected to result in a higher level of functional redundancy, and hence, more resistance and resilience to disturbances [93]. Increased abundance of genes relating to sporulation was predicted in *C*. *pallens* soils; this could also indicate greater functional redundancy in those soils.

Although total bacterial and nitrogen-fixing communities differed between soils, the proportion of the bacterial community that was diazotrophic was similar. In contrast to the total bacterial community, the diversity of the diazotrophic community was greater in the *C*. *teretifolia* soils, potentially increasing resilience of the diazotroph community in this soil. This highlights the importance of understanding the ecological role and responses of different communities to a changing environment, particularly in these habitats where alpine plant biodiversity is vulnerable to the potential effects of climate change, with models predicting a 33–50% loss of indigenous alpine species following a 3°C temperature rise [94]. Microbial communities play important roles in nutrient cycling in soils [95] and have a significant impact on the productivity and diversity of plant communities [96]. Elevated temperature altered bacterial community composition and specifically diazotroph community structure in prairie grasslands [97,98]. Furthermore, changes in soil diazotroph community structure can impact on nitrogen fixation rates [99]. In this study, extensive differences were observed in total bacterial and diazotrophic communities between two sites which are geographically close and dominated by grasses belonging to the same genus. We predict these differences will alter the response of the communities to environmental change.

## Supporting information

S1 Fig Rarefaction curves of sub-sampled datasets indicating the observed number of OTUs within the 198 769 random 16S rRNA gene sequences (a and b) and 9658 random *nifH* gene sequences (c and d). Clustering was performed at 97% similarity cut-off.
(TIF)

S2 Fig Bayesian phylogenetic tree inferred from 97% nucleotide similarity clustered 16S rRNA gene sequences from the class *Ktedonobacteria* (*Chloroflexi*) and their nearest relatives. 16S rRNA gene of *Chloroflexus aggregans* was used as an outgroup. Heatmap illustrating the relative number of sequences for each OTU is shown, with the lower values of the dataset coloured in blue and the highest value in bright red. Numbers associated with nodes are Bayesian posterior probabilities. GenBank accession numbers are indicated next to reference taxa. For clarity purposes only OTUs with 100 sequences and more have been included in this tree.
(TIF)

S3 Fig Bayesian phylogenetic tree inferred from 97% nucleotide similarity clustered 16S rRNA gene sequences from the family *Nitrosomonadaceae* (*Betaproteobacteria*) and their nearest relatives. 16S rRNA gene of *Nitrosococcus oceani* (*Gammaproteobacteria*) was used as an outgroup. (Numbers associated with nodes are Bayesian posterior probabilities. GenBank accession numbers are indicated next to reference taxa. For clarity purposes only OTUs with 100 sequences and more have been included in this tree.
(TIF)

S4 Fig Proportional abundances of *nifH* per 1000 copies of 16S rRNA gene in *Chionochloa pallens* (CP) and *Chionochloa teretifolia* (CT) grassland soil samples, as quantified by real-time quantitative PCR. (PDF)

S1 Table Nucleotide sequences of primers used in the construction of libraries for Illumina sequencing. Lowercase letters indicate adapter sequences required for binding to the flow cell, underlined lowercase indicate binding sites for the Illumina sequencing primers, bold uppercase indicate the Illumina TruSeq 6-bp index sequence, and regular uppercase are the 16S rRNA and *nifH* gene primers 0341F/0781R (Klindworth et al., 2013) and 19F/407R (Ueda et al., 1995), respectively.
(DOCX)

S2 Table Number of sequences before and after initial processing and filtering steps. (DOCX)

S3 Table Bray-Curtis distances between 16S rRNA and *nifH* genes-based bacterial community samples from *C*. *pallens* and *C*. *teretifolia* soils. (DOCX)

S4 Table Relative abundances of bacterial phyla and proteobacterial classes in *Chionochloa pallens* and *Chionochloa teretifolia* grassland soils. *P < 0.1, **P < 0.05 and ***P < 0.001 denote significant differences between *C*. *pallens* and *C*. *teretifolia* samples (one-way **ANOVA**).
(DOCX)

S5 Table Abundance of KEGG orthologs (KO) and selected genes associated with sporulation predicted using Tax4Fun from 16S rRNA gene dataset. (DOCX)
