# Supplementary figures and images for: Contrasting bacterial communities in two indigenous Chionochloa (Poaceae) grassland soils in New Zealand
Source: PLoS One. 2017 Jun 28;12(6):e0179652. doi: 10.1371/journal.pone.0179652 (PMC5489180; doi:10.1371/journal.pone.0179652)

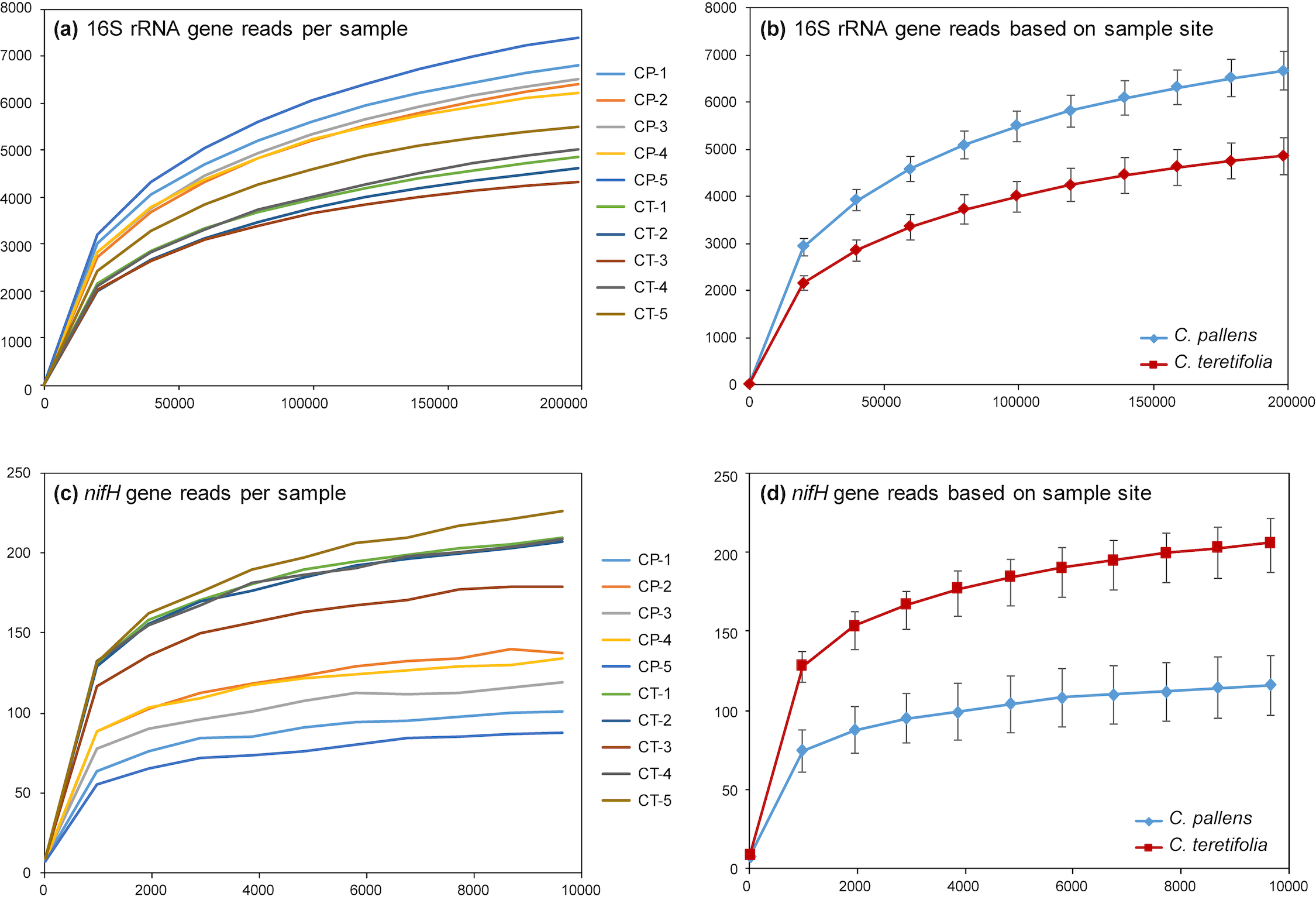

Supplement: S1 Fig — Clustering was performed at 97% similarity cut-off. (TIF) [file pone.0179652.s001.tif]

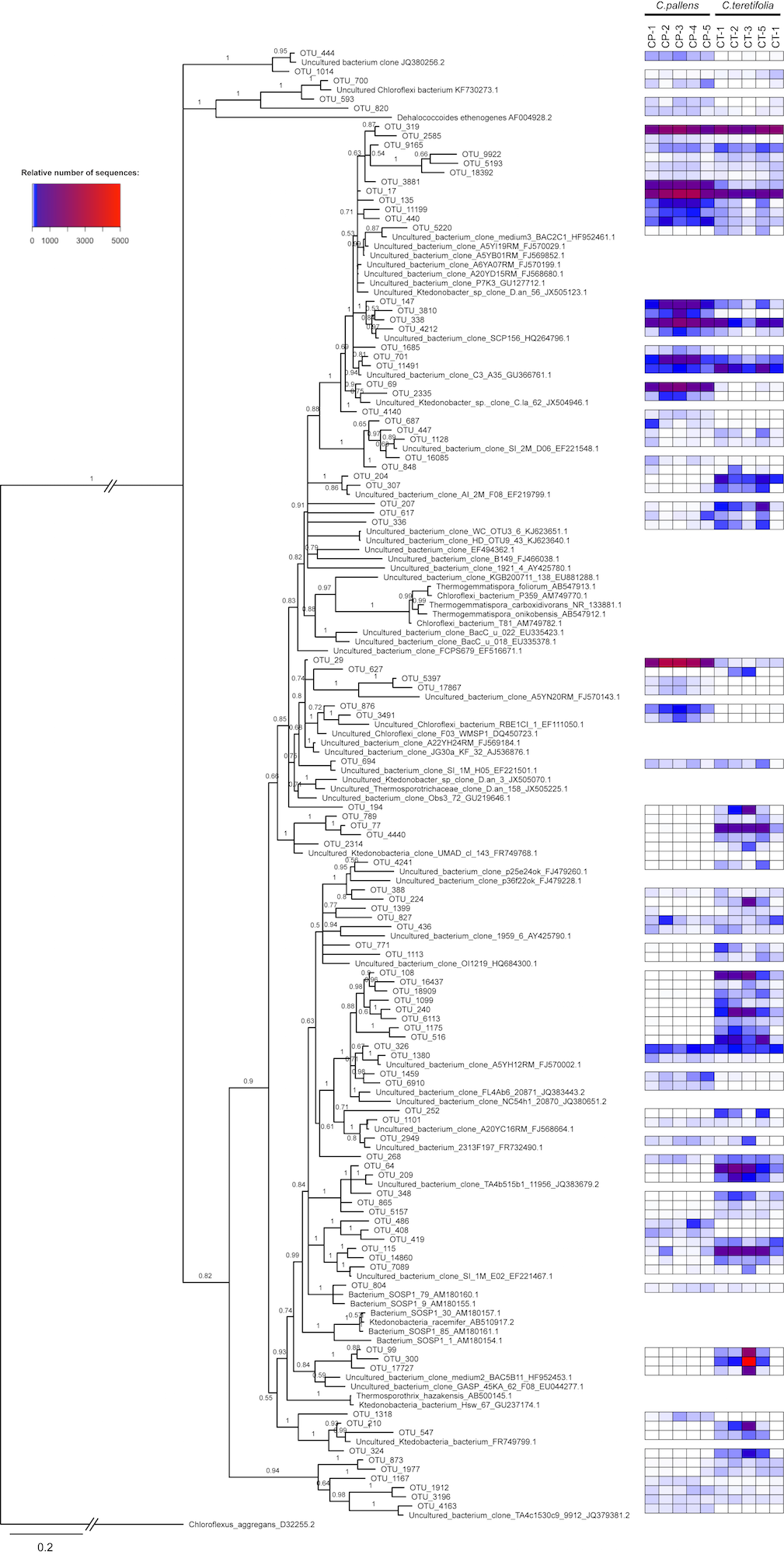

Supplement: S2 Fig — 16S rRNA gene of Chloroflexus aggregans was used as an outgroup. Heatmap illustrating the relative number of sequences for each OTU is shown, with the lower values of the dataset coloured in blue and the highest value in bright red. Numbers associated with nodes are Bayesian posterior probabilities. GenBank accession numbers are indicated next to reference taxa. For clarity purposes only OTUs with 100 sequences and more have been included in this tree. (TIF) [file pone.0179652.s002.tif]

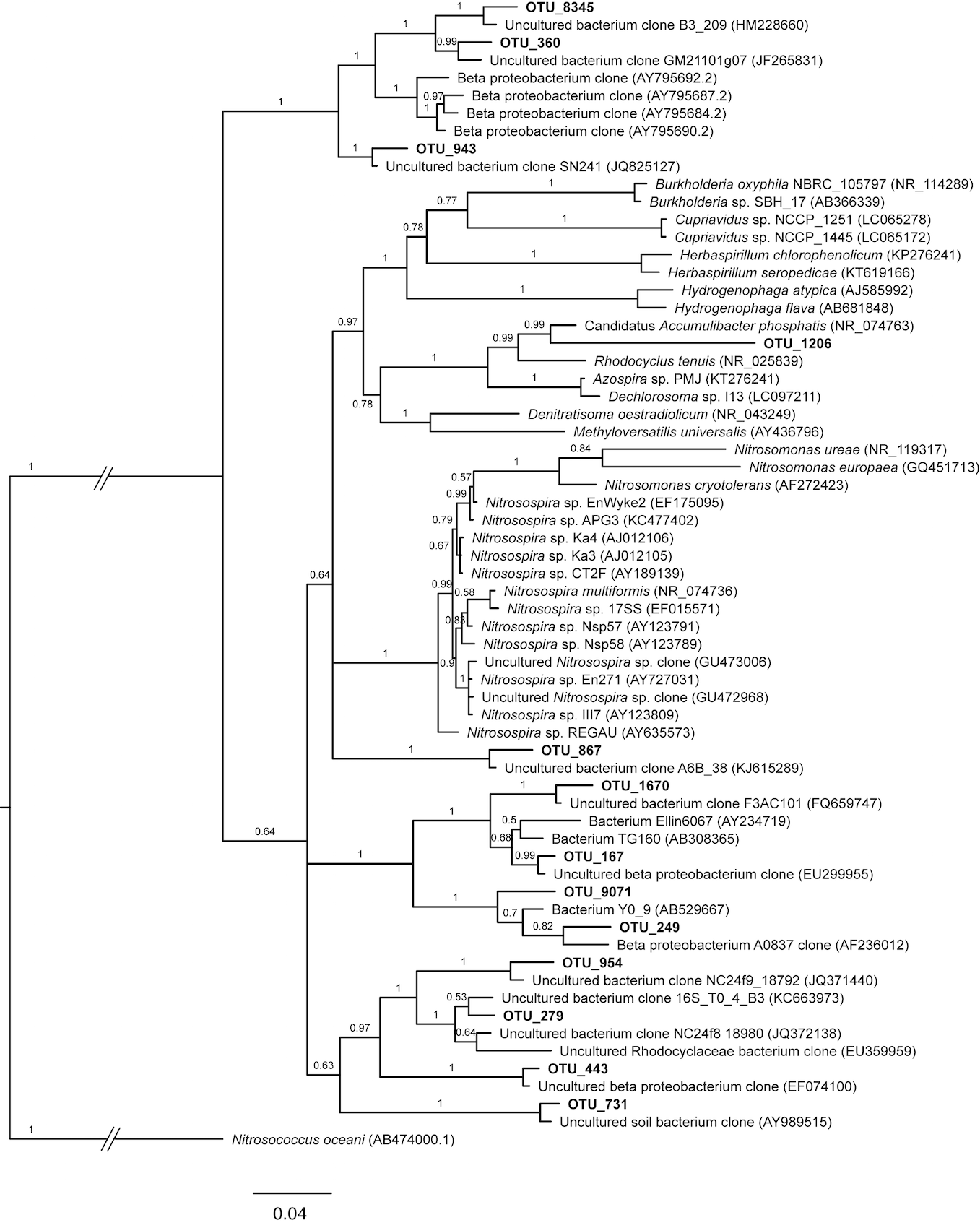

Supplement: S3 Fig — 16S rRNA gene of Nitrosococcus oceani (Gammaproteobacteria) was used as an outgroup. (Numbers associated with nodes are Bayesian posterior probabilities. GenBank accession numbers are indicated next to reference taxa. For clarity purposes only OTUs with 100 sequences and more have been included in this tree. (TIF) [file pone.0179652.s003.tif]

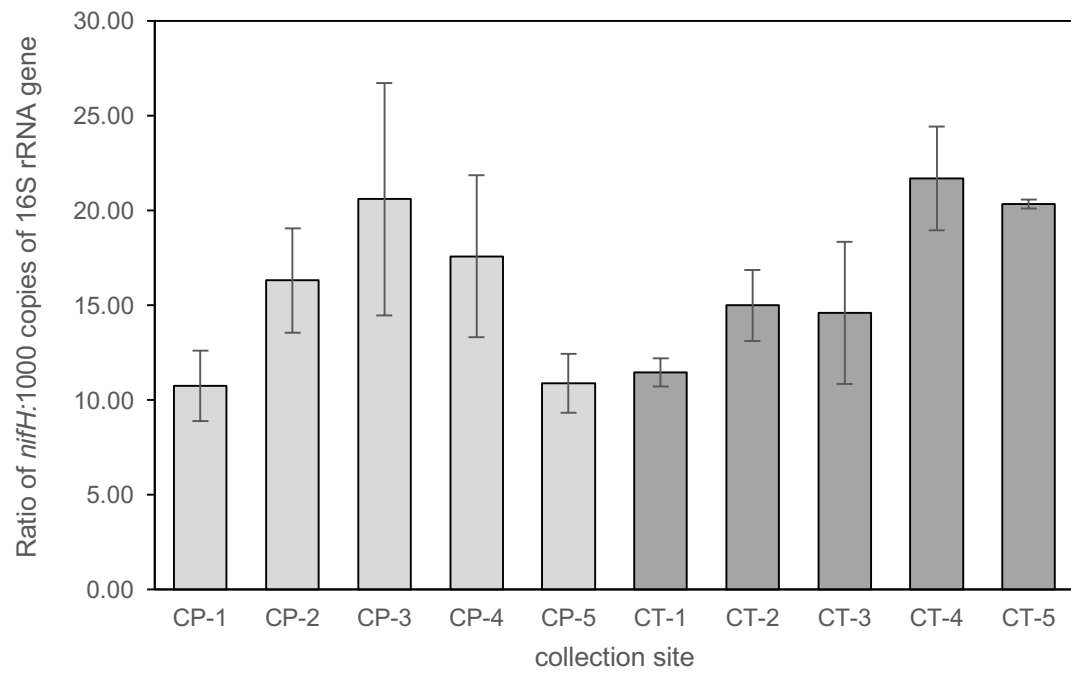

Supplement: S4 Fig — (PDF) [file pone.0179652.s004.pdf]
