# Supplementary material for: Contrasting bacterial communities in two indigenous Chionochloa (Poaceae) grassland soils in New Zealand
Source: PLoS One. 2017 Jun 28;12(6):e0179652. doi: 10.1371/journal.pone.0179652 (PMC5489180; doi:10.1371/journal.pone.0179652)
Supplement: S1 Table — Lowercase letters indicate adapter sequences required for binding to the flow cell, underlined lowercase indicate binding sites for the Illumina sequencing primers, bold uppercase indicate the Illumina TruSeq 6-bp index sequence, and regular uppercase are the 16S rRNA and nifH gene primers 0341F/0781R (Klindworth et al., 2013) and 19F/407R (Ueda et al., 1995), respectively. (DOCX) [file pone.0179652.s005.docx]

**Table S1.** **Nucleotide sequences of primers used in the construction of libraries for Illumina sequencing.** Lowercase letters indicate adapter sequences required for binding to the flow cell, underlined lowercase indicate binding sites for the Illumina sequencing primers, bold uppercase indicate the Illumina TruSeq 6-bp index sequence, and regular uppercase are the 16S rRNA and *nifH* gene primers 0341F/0781R (Klindworth et al., 2013) and 19F/407R (Ueda et al., 1995), respectively.

| **Name** | **Sequence (5’ – 3’)** |
| --- | --- |
| 0341F_modified | acgacgctcttccgatctCCTACGGGNGGCWGCAG |
| 0781R_modified | cgtgtgctcttccgatctGACTACHVGGGTATCTAATCC |
| 19F_modified | acgacgctcttccgatctGCIWTYTAYGGIAARGGIGG |
| 407R_modified | cgtgtgctcttccgatctAAICCRCCRCAIACIACRTC |
| CP_1_16S_R | caagcagaagacggcatacgagat**GTATAG**gtgactggagttcagacgtgtgctcttccgatc |
| CP_2_16S_R | caagcagaagacggcatacgagat**TCTGAG**gtgactggagttcagacgtgtgctcttccgatc |
| CP_3_16S_R | caagcagaagacggcatacgagat**GTCGTC**gtgactggagttcagacgtgtgctcttccgatc |
| CP_4_16S_R | caagcagaagacggcatacgagat**CGATTA**gtgactggagttcagacgtgtgctcttccgatc |
| CP_5_16S_R | caagcagaagacggcatacgagat**GCTGTA**gtgactggagttcagacgtgtgctcttccgatc |
| CT_1_16S_R | caagcagaagacggcatacgagat**ATTATA**gtgactggagttcagacgtgtgctcttccgatc |
| CT_2_16S_R | caagcagaagacggcatacgagat**GAATGA**gtgactggagttcagacgtgtgctcttccgatc |
| CT_3_16S_R | caagcagaagacggcatacgagat**TCGGGA**gtgactggagttcagacgtgtgctcttccgatc |
| CT_4_16S_R | caagcagaagacggcatacgagat**CTTCGA**gtgactggagttcagacgtgtgctcttccgatc |
| CT_5_16S_R | caagcagaagacggcatacgagat**TGCCGA**gtgactggagttcagacgtgtgctcttccgatc |
| CP_1_nifH_R | caagcagaagacggcatacgagat**GTAGCC**gtgactggagttcagacgtgtgctcttccgatc |
| CP_2_nifH_R | caagcagaagacggcatacgagat**TACAAG**gtgactggagttcagacgtgtgctcttccgatc |
| CP_3_nifH_R | caagcagaagacggcatacgagat**GTCGTC**gtgactggagttcagacgtgtgctcttccgatc |
| CP_4_nifH_R | caagcagaagacggcatacgagat**GGAACT**gtgactggagttcagacgtgtgctcttccgatc |
| CP_5_nifH_R | caagcagaagacggcatacgagat**TGACAT**gtgactggagttcagacgtgtgctcttccgatc |
| CT_1_nifH_R | caagcagaagacggcatacgagat**GGACGG**gtgactggagttcagacgtgtgctcttccgatc |
| CT_2_nifH_R | caagcagaagacggcatacgagat**CTCTAC**gtgactggagttcagacgtgtgctcttccgatc |
| CT_3_nifH_R | caagcagaagacggcatacgagat**GCGGAC**gtgactggagttcagacgtgtgctcttccgatc |
| CT_4_nifH_R | caagcagaagacggcatacgagat**TTTCAC**gtgactggagttcagacgtgtgctcttccgatc |
| CT_5_nifH_R | caagcagaagacggcatacgagat**GGCCAC**gtgactggagttcagacgtgtgctcttccgatc |
| TruSeq_F | aatgatacggcgaccaccgagatctacactctttccctacacgacgctcttccgatct |

**References**

Klindworth A., Pruesse E., Schweer T., Peplies J., Quast C., Horn M., Glöckner FO. 2013. Evaluation of general 16S ribosomal RNA gene PCR primers for classical and next-generation sequencing-based diversity studies. *Nucleic Acids Research* 41:1–11. DOI: 10.1093/nar/gks808.

Ueda T., Suga Y., Yahiro N., Matsuguchi T. 1995. Remarkable N_2_-fixing bacterial diversity detected in rice roots by molecular evolutionary analysis of *nifH* gene sequences. *Journal of bacteriology* 177:1414–1417.
