# Supplementary material for: Contrasting bacterial communities in two indigenous Chionochloa (Poaceae) grassland soils in New Zealand
Source: PLoS One. 2017 Jun 28;12(6):e0179652. doi: 10.1371/journal.pone.0179652 (PMC5489180; doi:10.1371/journal.pone.0179652)
Supplement: S2 Table — (DOCX) [file pone.0179652.s006.docx]

**Table S2.** **Number of sequences before and after initial processing and filtering steps.**

|  | **16S rRNA gene sequences** | | | ***nifH* gene sequences** | |
| --- | --- | --- | --- | --- | --- |
| **Sample** | **Paired-end reads** | **UPARSE filtering** | **Chimera and other filtering** | **Paired-end reads** | **UPARSE filtering** |
| CP-1 | 698,861 | 382,378 | 324,918 | 175,934 | 142,752 |
| CP-2 | 1,003,265 | 562,480 | 325,435 | 317,846 | 259,464 |
| CP-3 | 1,111,352 | 589,083 | 260,222 | 196,708 | 156,718 |
| CP-4 | 879,370 | 483,973 | 248,486 | 323,850 | 265,199 |
| CP-5 | 813,025 | 423,323 | 301,802 | 281,577 | 228,701 |
| CT-1 | 1,008,689 | 562,667 | 315,173 | 280,524 | 226,856 |
| CT-2 | 992,716 | 554,601 | 275,539 | 301,630 | 243,382 |
| CT-3 | 887,986 | 495,277 | 304,017 | 315,781 | 241,587 |
| CT-4 | 738,240 | 399,343 | 365,588 | 306,520 | 243,382 |
| CT-5 | 1,200,054 | 645,323 | 198,769 | 350,927 | 281,890 |
