# Supplementary material for: Contrasting bacterial communities in two indigenous Chionochloa (Poaceae) grassland soils in New Zealand
Source: PLoS One. 2017 Jun 28;12(6):e0179652. doi: 10.1371/journal.pone.0179652 (PMC5489180; doi:10.1371/journal.pone.0179652)
Supplement: S3 Table — (DOCX) [file pone.0179652.s007.docx]

**Table S3.** **Bray-Curtis distances between 16S rRNA and *nifH* gene-based bacterial community samples from *C. pallens* and *C. teretifolia* soils.**

| **16S rRNA gene** | | | | | | | | | | | |
| --- | --- | --- | --- | --- | --- | --- | --- | --- | --- | --- | --- |
|  |  | *C. pallens* samples | | | | | *C. teretifolia* samples | | | | |
|  |  | CP-1 | CP-2 | CP-3 | CP-4 | CP-5 | CT-1 | CT-2 | CT-3 | CT-4 | CT-5 |
| *C. pallens* samples | CP-1 | 0.0000 | 0.2406 | 0.2323 | 0.2132 | 0.2339 | 0.4933 | 0.5184 | 0.5376 | 0.5007 | 0.4573 |
|  | CP-2 | 0.2406 | 0.0000 | 0.1615 | 0.2299 | 0.3007 | 0.4709 | 0.4977 | 0.5297 | 0.4710 | 0.4252 |
|  | CP-3 | 0.2323 | 0.1615 | 0.0000 | 0.1901 | 0.2803 | 0.4671 | 0.4864 | 0.5265 | 0.4708 | 0.4240 |
|  | CP-4 | 0.2132 | 0.2299 | 0.1901 | 0.0000 | 0.2393 | 0.4981 | 0.5161 | 0.5487 | 0.5062 | 0.4671 |
|  | CP-5 | 0.2339 | 0.3007 | 0.2803 | 0.2393 | 0.0000 | 0.5585 | 0.5741 | 0.5955 | 0.5637 | 0.5211 |
| *C. teretifolia* samples | CT-1 | 0.4933 | 0.4709 | 0.4671 | 0.4981 | 0.5585 | 0.0000 | 0.1561 | 0.2457 | 0.1114 | 0.2016 |
|  | CT-2 | 0.5184 | 0.4977 | 0.4864 | 0.5161 | 0.5741 | 0.1561 | 0.0000 | 0.2216 | 0.1589 | 0.2526 |
|  | CT-3 | 0.5376 | 0.5297 | 0.5265 | 0.5487 | 0.5955 | 0.2457 | 0.2216 | 0.0000 | 0.2588 | 0.3271 |
|  | CT-4 | 0.5007 | 0.4710 | 0.4708 | 0.5062 | 0.5637 | 0.1114 | 0.1589 | 0.2588 | 0.0000 | 0.1831 |
|  | CT-5 | 0.4573 | 0.4252 | 0.4240 | 0.4671 | 0.5211 | 0.2016 | 0.2526 | 0.3271 | 0.1831 | 0.0000 |

| ***nifH* gene** | | | | | | | | | | | |
| --- | --- | --- | --- | --- | --- | --- | --- | --- | --- | --- | --- |
|  |  | CP-1 | CP-2 | CP-3 | CP-4 | CP-5 | CT-1 | CT-2 | CT-3 | CT-4 | CT-5 |
| *C. pallens* samples | CP-1 | 0.0000 | 0.5367 | 0.6361 | 0.6949 | 0.7780 | 0.8793 | 0.8809 | 0.9241 | 0.9088 | 0.8789 |
|  | CP-2 | 0.5367 | 0.0000 | 0.5594 | 0.7285 | 0.7799 | 0.8006 | 0.8446 | 0.8690 | 0.8272 | 0.8043 |
|  | CP-3 | 0.6361 | 0.5594 | 0.0000 | 0.6856 | 0.7244 | 0.8399 | 0.8486 | 0.8589 | 0.7969 | 0.8312 |
|  | CP-4 | 0.6949 | 0.7285 | 0.6856 | 0.0000 | 0.6585 | 0.8851 | 0.8830 | 0.9019 | 0.8788 | 0.8798 |
|  | CP-5 | 0.7780 | 0.7799 | 0.7244 | 0.6585 | 0.0000 | 0.8899 | 0.8789 | 0.9065 | 0.8769 | 0.8802 |
| *C. teretifolia* samples | CT-1 | 0.8793 | 0.8006 | 0.8399 | 0.8851 | 0.8899 | 0.0000 | 0.4232 | 0.5800 | 0.4369 | 0.3498 |
|  | CT-2 | 0.8809 | 0.8446 | 0.8486 | 0.8830 | 0.8789 | 0.4232 | 0.0000 | 0.4811 | 0.3607 | 0.3955 |
|  | CT-3 | 0.9241 | 0.8690 | 0.8589 | 0.9019 | 0.9065 | 0.5800 | 0.4811 | 0.0000 | 0.5232 | 0.5659 |
|  | CT-4 | 0.9088 | 0.8272 | 0.7969 | 0.8788 | 0.8769 | 0.4369 | 0.3607 | 0.5232 | 0.0000 | 0.3577 |
|  | CT-5 | 0.8789 | 0.8043 | 0.8312 | 0.8798 | 0.8802 | 0.3498 | 0.3955 | 0.5659 | 0.3577 | 0.0000 |
