# Supplementary material for: Contrasting bacterial communities in two indigenous Chionochloa (Poaceae) grassland soils in New Zealand
Source: PLoS One. 2017 Jun 28;12(6):e0179652. doi: 10.1371/journal.pone.0179652 (PMC5489180; doi:10.1371/journal.pone.0179652)
Supplement: S4 Table — *P < 0.1, **P < 0.05 and ***P < 0.001 denote significant differences between C. pallens and C. teretifolia samples (one-way ANOVA). (DOCX) [file pone.0179652.s008.docx]

**Table S4.** **Relative abundances of bacterial phyla and Proteobacterial classes in *Chionochloa pallens* and *Chionochloa teretifolia* grassland soils.** **P* < 0.1, ***P* < 0.05 and ****P* < 0.001 denote significant differences between *C. pallens* and *C. teretifolia* samples (one-way anova).

|  | **Relative abundance (%)** | | | | | | | | | | | |
| --- | --- | --- | --- | --- | --- | --- | --- | --- | --- | --- | --- | --- |
|  | ***C. pallens*** | | | | | | ***C. teretifolia*** | | | | | |
| **Phylum** | **CP-1** | **CP-2** | **CP-3** | **CP-4** | **CP-5** | **Mean ± SD** | **CT-1** | **CT-2** | **CT-3** | **CT-4** | **CT-5** | **Mean ± SD** |
| *Acidobacteria**** | 22.69 | 24.32 | 24.40 | 22.62 | 18.81 | 22.57 ± 2.03 | 31.21 | 30.94 | 29.08 | 32.99 | 37.19 | 32.28 ± 2.75 |
| *Actinobacteria*** | 8.29 | 6.86 | 7.74 | 9.95 | 7.72 | 8.11 ± 1.02 | 13.47 | 18.18 | 16.91 | 13.30 | 6.96 | 13.76 ± 3.90 |
| *Armatimonadetes* | 0.54 | 0.37 | 0.45 | 0.53 | 0.43 | 0.46 ± 0.06 | 0.51 | 0.35 | 0.65 | 0.41 | 0.46 | 0.48 ± 0.10 |
| *Bacteroidetes**** | 7.31 | 5.35 | 5.49 | 5.87 | 6.71 | 6.15 ± 0.75 | 3.06 | 2.22 | 2.65 | 2.78 | 3.21 | 2.78 ± 0.34 |
| BD1-5*** | 0.01 | 0.01 | 0.01 | 0.01 | 0.01 | 0.01 ± 0.00 | 0.00 | 0.00 | 0.00 | 0.00 | 0.00 | 0.00 ± 0.00 |
| BHI80-139 | 0.01 | 0.01 | 0.02 | 0.01 | 0.01 | 0.01 ± 0.00 | 0.01 | 0.01 | 0.01 | 0.01 | 0.01 | 0.01 ± 0.00 |
| Candidate division OD1** | 0.48 | 0.30 | 0.22 | 0.33 | 0.45 | 0.36 ± 0.10 | 0.10 | 0.07 | 0.11 | 0.11 | 0.20 | 0.12 ± 0.04 |
| Candidate division TM7** | 1.45 | 0.93 | 0.94 | 0.97 | 2.40 | 1.34 ± 0.57 | 0.69 | 0.52 | 0.63 | 0.46 | 0.60 | 0.58 ± 0.08 |
| Candidate division WS3** | 0.14 | 0.23 | 0.12 | 0.05 | 0.13 | 0.13 ± 0.06 | 0.00 | 0.00 | 0.00 | 0.00 | 0.00 | 0.00 ± 0.00 |
| *Chlamydiae* | 0.41 | 0.59 | 0.33 | 0.30 | 0.56 | 0.44 ± 0.12 | 0.39 | 0.22 | 0.29 | 0.38 | 0.38 | 0.33 ± 0.07 |
| *Chlorobi**** | 0.21 | 0.25 | 0.17 | 0.19 | 0.18 | 0.20 ± 0.03 | 0.05 | 0.03 | 0.05 | 0.05 | 0.05 | 0.04 ± 0.01 |
| *Chloroflexi*** | 6.64 | 9.19 | 10.47 | 8.96 | 6.72 | 8.39 ± 1.49 | 5.32 | 5.29 | 8.91 | 5.13 | 3.43 | 5.62 ± 1.79 |
| *Cyanobacteria* | 0.45 | 0.19 | 0.28 | 0.52 | 0.64 | 0.42 ± 0.16 | 0.50 | 0.85 | 0.39 | 0.75 | 0.36 | 0.57 ± 0.20 |
| *Elusimicrobia*** | 0.34 | 0.26 | 0.22 | 0.20 | 0.27 | 0.26 ± 0.05 | 0.14 | 0.12 | 0.20 | 0.15 | 0.22 | 0.17 ± 0.03 |
| *Fibrobacteres*** | 0.16 | 0.06 | 0.05 | 0.07 | 0.06 | 0.08 ± 0.03 | 0.00 | 0.01 | 0.01 | 0.01 | 0.01 | 0.07 ± 0.03 |
| *Firmicutes**** | 0.20 | 0.21 | 0.21 | 0.27 | 0.17 | 0.21 ± 0.03 | 0.05 | 0.05 | 0.03 | 0.04 | 0.08 | 0.05 ± 0.02 |
| *Gemmatimonadetes**** | 0.69 | 0.61 | 0.41 | 0.49 | 0.60 | 0.56 ± 0.10 | 0.10 | 0.09 | 0.06 | 0.10 | 0.11 | 0.09 ± 0.02 |
| *Nitrospirae* | 0.08 | 0.14 | 0.09 | 0.03 | 0.06 | 0.08 ± 0.03 | 0.09 | 0.05 | 0.04 | 0.07 | 0.11 | 0.07 ± 0.03 |
| Other and unclassified*** | 0.72 | 0.77 | 0.87 | 0.72 | 0.89 | 0.79 ± 0.07 | 1.05 | 1.14 | 1.36 | 1.11 | 1.21 | 1.17 ± 0.11 |
| *Planctomycetes*** | 8.55 | 8.48 | 8.30 | 7.55 | 9.72 | 8.52 ± 0.70 | 6.87 | 6.31 | 7.22 | 7.15 | 7.97 | 7.10 ± 0.54 |
| α-*Proteobacteria*** | 19.91 | 22.22 | 22.08 | 22.97 | 23.67 | 22.17 ± 1.26 | 19.26 | 19.35 | 16.97 | 20.13 | 20.74 | 19.29 ± 1.28 |
| β-*Proteobacteria**** | 3.53 | 3.15 | 2.80 | 2.91 | 3.19 | 3.12 ± 0.25 | 2.07 | 0.99 | 0.98 | 1.31 | 1.58 | 1.39 ± 0.41 |
| δ-*Proteobacteria*** | 2.89 | 1.90 | 1.82 | 2.12 | 2.28 | 2.20 ± 0.38 | 1.62 | 1.37 | 1.20 | 1.50 | 1.82 | 1.50 ± 0.21 |
| γ-*Proteobacteria*** | 5.80 | 5.87 | 5.13 | 5.24 | 4.77 | 5.36 ± 0.42 | 7.74 | 6.45 | 6.09 | 5.95 | 6.15 | 6.48 ± 0.65 |
| Other *Proteobacteria* | 0.76 | 0.81 | 0.66 | 0.66 | 0.70 | 0.70 ± 0.07 | 0.74 | 0.82 | 0.84 | 0.73 | 0.71 | 0.76 ± 0.06 |
| SHA-109 | 0.05 | 0.03 | 0.06 | 0.04 | 0.03 | 0.04 ± 0.01 | 0.05 | 0.04 | 0.07 | 0.05 | 0.07 | 0.06 ± 0.01 |
| SM2F11** | 0.08 | 0.10 | 0.15 | 0.15 | 0.09 | 0.11 ± 0.03 | 0.05 | 0.04 | 0.03 | 0.05 | 0.06 | 0.05 ± 0.01 |
| *Spirochaetae* | 0.16 | 0.04 | 0.01 | 0.02 | 0.04 | 0.05 ± 0.05 | 0.01 | 0.00 | 0.00 | 0.01 | 0.01 | 0.01 ± 0.00 |
| TM6* | 0.08 | 0.15 | 0.11 | 0.09 | 0.15 | 0.11 ± 0.03 | 0.08 | 0.05 | 0.03 | 0.08 | 0.12 | 0.07 ± 0.03 |
| *Verrucomicrobia*** | 6.32 | 5.36 | 5.38 | 5.20 | 7.48 | 5.95 ± 0.87 | 3.39 | 2.97 | 2.79 | 3.40 | 4.83 | 3.47 ± 0.72 |
| WCHB1-60 | 0.27 | 0.15 | 0.17 | 0.27 | 0.37 | 0.25 ± 0.08 | 0.10 | 0.12 | 0.52 | 0.09 | 0.14 | 0.19 ± 0.17 |
| WD272** | 0.78 | 1.09 | 0.84 | 0.69 | 0.69 | 0.82 ± 0.15 | 1.28 | 1.35 | 1.88 | 1.70 | 1.21 | 1.49 ± 0.26 |
