# Supplementary material for: Contrasting bacterial communities in two indigenous Chionochloa (Poaceae) grassland soils in New Zealand
Source: PLoS One. 2017 Jun 28;12(6):e0179652. doi: 10.1371/journal.pone.0179652 (PMC5489180; doi:10.1371/journal.pone.0179652)
Supplement: S5 Table — (DOCX) [file pone.0179652.s009.docx]

**Table S5. Abundance of KEGG Orthologs (KO) and selected genes associated with sporulation predicted using Tax4Fun from 16S rRNA gene dataset.**

| **KO** | **Description** | **CP-1** | **CP-2** | **CP-3** | **CP-4** | **CP-5** | **CT-1** | **CT-2** | **CT-3** | **CT-4** | **CT-5** | **Mean CP** | **Mean CT** |
| --- | --- | --- | --- | --- | --- | --- | --- | --- | --- | --- | --- | --- | --- |
| K04769 | AbrB family transcriptional regulator, stage V sporulation protein T | 9.09E-06 | 8.53E-06 | 1.17E-05 | 1.62E-05 | 7.08E-06 | 1.03E-06 | 5.77E-07 | 5.76E-07 | 5.36E-07 | 1.21E-06 | 1.1E-05 | 7.9E-07 |
| K06283 | putative DeoR family transcriptional regulator, stage III sporulation protein D | 4.03E-06 | 3.60E-06 | 5.18E-06 | 7.67E-06 | 2.83E-06 | 5.66E-07 | 2.84E-07 | 2.57E-07 | 2.32E-07 | 6.06E-07 | 4.7E-06 | 3.9E-07 |
| K06284 | transcriptional pleiotropic regulator of transition state genes | 5.99E-06 | 5.54E-06 | 7.29E-06 | 1.01E-05 | 4.91E-06 | 9.73E-07 | 4.59E-07 | 3.80E-07 | 4.66E-07 | 1.04E-06 | 6.8E-06 | 6.6E-07 |
| K06285 | transcription attenuation protein (tryptophan RNA-binding attenuator protein) | 9.68E-07 | 8.81E-07 | 1.11E-06 | 1.44E-06 | 1.05E-06 | 7.91E-08 | 8.68E-08 | 8.26E-08 | 1.12E-07 | 1.65E-07 | 1.1E-06 | 1.1E-07 |
| K06289 | spore germination protein AB | 3.58E-07 | 2.64E-07 | 4.33E-07 | 4.44E-07 | 6.56E-07 | 1.82E-08 | 1.62E-08 | 0 | 8.82E-08 | 2.07E-08 | 4.3E-07 | 2.9E-08 |
| K06290 | spore germination protein AC | 5.76E-07 | 4.87E-07 | 7.33E-07 | 8.51E-07 | 8.26E-07 | 2.26E-08 | 2.68E-08 | 1.33E-08 | 9.94E-08 | 4.24E-08 | 6.9E-07 | 4.1E-08 |
| K06291 | spore germination protein BA | 5.40E-09 | 3.23E-09 | 4.75E-09 | 4.92E-09 | 5.66E-09 | 6.24E-11 | 2.19E-10 | 0 | 7.99E-10 | 2.72E-10 | 4.8E-09 | 2.7E-10 |
| K06292 | spore germination protein BB | 2.09E-07 | 1.66E-07 | 2.64E-07 | 2.79E-07 | 4.70E-07 | 8.54E-09 | 7.96E-09 | 0 | 6.61E-08 | 6.65E-09 | 2.8E-07 | 1.8E-08 |
| K06293 | spore germination protein BC | 2.44E-07 | 1.85E-07 | 2.91E-07 | 3.06E-07 | 4.92E-07 | 8.47E-09 | 9.45E-09 | 0 | 6.86E-08 | 8.51E-09 | 3.0E-07 | 1.9E-08 |
| K06296 | spore germination protein KB | 6.33E-06 | 6.01E-06 | 7.36E-06 | 8.73E-06 | 6.83E-06 | 5.59E-07 | 4.55E-07 | 4.90E-07 | 7.11E-07 | 9.56E-07 | 7.1E-06 | 6.3E-07 |
| K06313 | spore germination protein | 8.90E-06 | 7.76E-06 | 1.16E-05 | 1.34E-05 | 7.91E-06 | 9.25E-07 | 2.66E-07 | 2.34E-07 | 6.92E-07 | 1.41E-06 | 9.9E-06 | 7.1E-07 |
| K06315 | transcriptional regulator of the spore photoproduct lyase operon | 7.02E-08 | 5.09E-08 | 8.51E-08 | 8.42E-08 | 1.28E-07 | 4.45E-09 | 2.71E-09 | 0 | 1.74E-08 | 7.82E-09 | 8.4E-08 | 6.5E-09 |
| K06317 | inhibitor of the pro-sigma K processing machinery | 2.41E-06 | 2.21E-06 | 3.31E-06 | 4.60E-06 | 1.73E-06 | 2.84E-07 | 1.11E-07 | 7.75E-08 | 1.19E-07 | 2.97E-07 | 2.9E-06 | 1.8E-07 |
| K06319 | spore maturation protein CgeA | 9.22E-08 | 7.01E-08 | 1.12E-07 | 1.17E-07 | 1.88E-07 | 5.06E-09 | 3.53E-09 | 0 | 2.63E-08 | 5.49E-09 | 1.2E-07 | 8.1E-09 |
| K06321 | spore maturation protein CgeC | 3.66E-08 | 3.22E-08 | 5.23E-08 | 5.59E-08 | 1.02E-07 | 2.05E-09 | 1.35E-09 | 0 | 1.48E-08 | 9.43E-10 | 5.6E-08 | 3.8E-09 |
| K06326 | spore coat protein C | 7.64E-09 | 5.67E-09 | 8.91E-09 | 9.33E-09 | 1.46E-08 | 2.27E-10 | 2.99E-10 | 0 | 1.93E-09 | 2.23E-10 | 9.2E-09 | 5.4E-10 |
| K06337 | spore coat-associated protein S | 4.86E-07 | 4.39E-07 | 6.12E-07 | 9.03E-07 | 4.16E-07 | 4.92E-08 | 3.23E-08 | 2.12E-08 | 4.12E-08 | 8.27E-08 | 5.7E-07 | 4.5E-08 |
| K06339 | spore coat protein T | 1.86E-08 | 1.45E-08 | 2.58E-08 | 2.42E-08 | 3.98E-08 | 6.97E-10 | 7.17E-10 | 0 | 5.53E-09 | 9.14E-10 | 2.5E-08 | 1.6E-09 |
| K06340 | spore coat protein V | 8.19E-08 | 6.38E-08 | 1.06E-07 | 1.07E-07 | 1.76E-07 | 3.14E-09 | 3.15E-09 | 0 | 2.47E-08 | 4.28E-09 | 1.1E-07 | 7.1E-09 |
| K06348 | sporulation inhibitor KapD | 2.08E-06 | 1.72E-06 | 2.88E-06 | 3.43E-06 | 1.62E-06 | 2.84E-07 | 3.21E-08 | 0 | 1.44E-07 | 4.01E-07 | 2.3E-06 | 1.7E-07 |
| K06352 | phosphatase RapA inhibitor | 2.02E-08 | 1.58E-08 | 2.51E-08 | 2.65E-08 | 4.38E-08 | 7.51E-10 | 7.77E-10 | 0 | 5.96E-09 | 6.70E-10 | 2.6E-08 | 1.6E-09 |
| K06353 | phosphatase RapC regulator | 6.46E-09 | 5.75E-09 | 9.36E-09 | 9.99E-09 | 1.84E-08 | 3.65E-10 | 2.39E-10 | 0 | 2.63E-09 | 0 | 1.0E-08 | 6.5E-10 |
| K06355 | phosphatase RapF regulator | 3.43E-09 | 3.57E-09 | 5.97E-09 | 6.45E-09 | 1.31E-08 | 2.88E-10 | 1.19E-10 | 0 | 1.93E-09 | 0 | 6.5E-09 | 4.7E-10 |
| K06359 | response regulator aspartate phosphatase A (stage 0 sporulation protein L) | 5.36E-07 | 3.87E-07 | 6.39E-07 | 6.38E-07 | 9.69E-07 | 2.99E-08 | 2.07E-08 | 0 | 1.34E-07 | 5.36E-08 | 6.3E-07 | 4.8E-08 |
| K06360 | response regulator aspartate phosphatase B | 1.51E-07 | 1.27E-07 | 2.07E-07 | 2.18E-07 | 3.85E-07 | 7.25E-09 | 5.67E-09 | 0 | 5.40E-08 | 4.27E-09 | 2.2E-07 | 1.4E-08 |
| K06362 | response regulator aspartate phosphatase D | 2.47E-07 | 2.28E-07 | 3.69E-07 | 3.88E-07 | 5.24E-07 | 1.51E-08 | 9.35E-09 | 0 | 6.98E-08 | 1.57E-08 | 3.5E-07 | 2.2E-08 |
| K06364 | response regulator aspartate phosphatase F | 1.97E-07 | 1.61E-07 | 2.59E-07 | 2.75E-07 | 4.74E-07 | 1.04E-08 | 7.43E-09 | 0 | 6.60E-08 | 8.03E-09 | 2.7E-07 | 1.8E-08 |
| K06366 | response regulator aspartate phosphatase H | 3.03E-07 | 2.14E-07 | 3.42E-07 | 3.48E-07 | 5.18E-07 | 8.75E-09 | 1.19E-08 | 0 | 6.99E-08 | 1.32E-08 | 3.5E-07 | 2.1E-08 |
| K06367 | response regulator aspartate phosphatase I | 4.61E-09 | 3.47E-09 | 7.01E-09 | 5.74E-09 | 9.12E-09 | 1.46E-10 | 1.79E-10 | 0 | 1.22E-09 | 6.90E-10 | 6.0E-09 | 4.5E-10 |
| K06368 | response regulator aspartate phosphatase J | 1.99E-07 | 1.62E-07 | 2.68E-07 | 2.76E-07 | 4.74E-07 | 1.09E-08 | 7.46E-09 | 0 | 6.69E-08 | 1.14E-08 | 2.8E-07 | 1.9E-08 |
| K06369 | response regulator aspartate phosphatase K | 1.52E-07 | 1.17E-07 | 2.00E-07 | 1.96E-07 | 3.20E-07 | 5.47E-09 | 5.86E-09 | 0 | 4.39E-08 | 1.17E-08 | 2.0E-07 | 1.3E-08 |
| K06372 | antagonist of SinR | 1.96E-08 | 1.80E-08 | 3.10E-08 | 3.15E-08 | 5.93E-08 | 1.20E-09 | 7.17E-10 | 0 | 8.53E-09 | 9.14E-10 | 3.2E-08 | 2.3E-09 |
| K06376 | stage 0 sporulation regulatory protein | 5.02E-08 | 3.88E-08 | 6.14E-08 | 6.48E-08 | 1.06E-07 | 1.87E-09 | 1.93E-09 | 0 | 1.48E-08 | 1.66E-09 | 6.4E-08 | 4.1E-09 |
| K06388 | stage II sporulation protein SA | 1.79E-07 | 1.58E-07 | 3.57E-07 | 2.74E-07 | 5.05E-07 | 1.04E-08 | 6.59E-09 | 0 | 7.42E-08 | 1.22E-08 | 2.9E-07 | 2.1E-08 |
| K06389 | stage II sporulation protein SB | 2.87E-08 | 2.17E-08 | 3.57E-08 | 3.59E-08 | 5.75E-08 | 9.64E-10 | 1.11E-09 | 0 | 7.89E-09 | 1.41E-09 | 3.6E-08 | 2.3E-09 |
| K06391 | stage III sporulation protein AB | 5.13E-06 | 4.77E-06 | 6.83E-06 | 8.91E-06 | 3.96E-06 | 6.58E-07 | 3.06E-07 | 2.66E-07 | 3.94E-07 | 7.32E-07 | 5.9E-06 | 4.7E-07 |
| K06392 | stage III sporulation protein AC | 2.36E-06 | 2.16E-06 | 2.95E-06 | 4.61E-06 | 1.69E-06 | 3.72E-07 | 2.01E-07 | 1.78E-07 | 1.48E-07 | 3.69E-07 | 2.8E-06 | 2.5E-07 |
| K06396 | stage III sporulation protein AG | 4.88E-06 | 4.39E-06 | 6.34E-06 | 8.95E-06 | 3.66E-06 | 7.83E-07 | 3.09E-07 | 2.53E-07 | 3.21E-07 | 7.61E-07 | 5.6E-06 | 4.9E-07 |
| K06405 | stage V sporulation protein AC | 8.39E-06 | 7.56E-06 | 1.07E-05 | 1.50E-05 | 6.14E-06 | 1.35E-06 | 5.93E-07 | 5.38E-07 | 6.35E-07 | 1.45E-06 | 9.6E-06 | 9.1E-07 |
| K06407 | stage V sporulation protein AE | 8.07E-06 | 7.09E-06 | 1.02E-05 | 1.49E-05 | 6.22E-06 | 1.30E-06 | 5.87E-07 | 4.67E-07 | 5.79E-07 | 1.58E-06 | 9.3E-06 | 9.0E-07 |
| K06421 | small acid-soluble spore protein D (minor alpha/beta-type SASP) | 1.44E-06 | 1.25E-06 | 1.91E-06 | 2.21E-06 | 1.32E-06 | 1.99E-07 | 5.41E-08 | 4.28E-08 | 1.17E-07 | 2.38E-07 | 1.6E-06 | 1.3E-07 |
| K06427 | small acid-soluble spore protein J (minor) | 3.13E-08 | 2.33E-08 | 3.80E-08 | 3.83E-08 | 6.05E-08 | 1.02E-09 | 1.21E-09 | 0 | 8.43E-09 | 1.68E-09 | 3.8E-08 | 2.5E-09 |
| K06429 | small acid-soluble spore protein L (minor) | 4.45E-08 | 3.42E-08 | 6.74E-08 | 5.72E-08 | 9.57E-08 | 2.11E-09 | 1.68E-09 | 0 | 1.58E-08 | 2.90E-09 | 6.0E-08 | 4.5E-09 |
| K06430 | small acid-soluble spore protein M (minor) | 2.12E-08 | 1.65E-08 | 2.76E-08 | 2.75E-08 | 4.54E-08 | 8.03E-10 | 8.16E-10 | 0 | 6.35E-09 | 1.19E-09 | 2.8E-08 | 1.8E-09 |
| K06434 | small acid-soluble spore protein (thioredoxin-like protein) | 1.20E-06 | 1.02E-06 | 1.50E-06 | 1.80E-06 | 1.11E-06 | 1.53E-07 | 5.50E-08 | 3.86E-08 | 1.07E-07 | 1.89E-07 | 1.3E-06 | 1.1E-07 |
| K06435 | required for translation of spoIIID | 8.06E-09 | 7.12E-09 | 1.31E-08 | 1.23E-08 | 2.26E-08 | 4.45E-10 | 2.99E-10 | 0 | 3.22E-09 | 6.90E-10 | 1.3E-08 | 9.3E-10 |
| K06436 | spore coat assemly protein | 9.85E-06 | 9.56E-06 | 1.31E-05 | 1.85E-05 | 7.37E-06 | 1.20E-06 | 6.57E-07 | 5.86E-07 | 6.02E-07 | 1.26E-06 | 1.2E-05 | 8.6E-07 |
| K06437 | sigma-E controlled sporulation protein | 1.61E-07 | 1.37E-07 | 2.37E-07 | 2.34E-07 | 4.15E-07 | 8.03E-09 | 6.05E-09 | 0 | 5.93E-08 | 9.85E-09 | 2.4E-07 | 1.7E-08 |
| K06438 | similar to stage IV sporulation protein | 1.33E-05 | 1.22E-05 | 1.72E-05 | 2.31E-05 | 1.03E-05 | 2.05E-06 | 1.02E-06 | 9.31E-07 | 1.20E-06 | 2.18E-06 | 1.5E-05 | 1.5E-06 |
| K06439 | similar to spore coat protein | 7.27E-07 | 6.57E-07 | 7.86E-07 | 8.06E-07 | 9.14E-07 | 6.89E-08 | 3.92E-08 | 5.10E-08 | 8.98E-08 | 1.12E-07 | 7.8E-07 | 7.2E-08 |
